# Specific amino acid supplementation rescues the heart from lipid overload-induced insulin resistance and contractile dysfunction by targeting the endosomal mTOR–v-ATPase axis

**DOI:** 10.1016/j.molmet.2021.101293

**Published:** 2021-07-13

**Authors:** Shujin Wang, Francesco Schianchi, Dietbert Neumann, Li-Yen Wong, Aomin Sun, Frans A. van Nieuwenhoven, Maurice P. Zeegers, Agnieszka Strzelecka, Umare Col, Jan F.C. Glatz, Miranda Nabben, Joost J.F.P. Luiken

**Affiliations:** 1 Department of Genetics & Cell Biology, Faculty of Health, Medicine and Life Sciences, Maastricht University, Maastricht, the Netherlands; 2 Institute of Life Sciences, Chongqing Medical University, Chongqing, PR China; 3 Department of Pathology, Maastricht University Medical Center+, Maastricht, the Netherlands; 4 Department of Clinical Genetics, Maastricht University Medical Center+, Maastricht, the Netherlands; 5 Department of Physiology, Faculty of Health, Medicine and Life Sciences, Maastricht University, Maastricht, the Netherlands; 6 CARIM School for Cardiovascular Diseases, Maastricht, the Netherlands; 7 Department of Complex Genetics and Epidemiology, School of Nutrition and Translational Research in Metabolism, Maastricht University, Maastricht, the Netherlands

**Keywords:** Vacuolar H^+^-ATPase, Endosomal CD36, mTORC1, Lipid-induced insulin resistance, Contractile function, Diabetic heart

## Abstract

**Objective:**

The diabetic heart is characterized by extensive lipid accumulation which often leads to cardiac contractile dysfunction. The underlying mechanism involves a pivotal role for vacuolar-type H^+^-ATPase (v-ATPase, functioning as endosomal/lysosomal proton pump). Specifically, lipid oversupply to the heart causes disassembly of v-ATPase and endosomal deacidification. Endosomes are storage compartments for lipid transporter CD36. However, upon endosomal deacidification, CD36 is expelled to translocate to the sarcolemma, thereby inducing myocardial lipid accumulation, insulin resistance, and contractile dysfunction. Hence, the v-ATPase assembly may be a suitable target for ameliorating diabetic cardiomyopathy. Another function of v-ATPase involves the binding of anabolic master-regulator mTORC1 to endosomes, a prerequisite for the activation of mTORC1 by amino acids (AAs). We examined whether the relationship between v-ATPase and mTORC1 also operates reciprocally; specifically, whether AA induces v-ATPase reassembly in a mTORC1-dependent manner to prevent excess lipids from entering and damaging the heart.

**Methods:**

Lipid overexposed rodent/human cardiomyocytes and high-fat diet-fed rats were treated with a specific cocktail of AAs (lysine/leucine/arginine). Then, v-ATPase assembly status/activity, cell surface CD36 content, myocellular lipid uptake/accumulation, insulin sensitivity, and contractile function were measured. To elucidate underlying mechanisms, specific gene knockdown was employed, followed by subcellular fractionation, and coimmunoprecipitation.

**Results:**

In lipid-overexposed cardiomyocytes, lysine/leucine/arginine reinternalized CD36 to the endosomes, prevented/reversed lipid accumulation, preserved/restored insulin sensitivity, and contractile function. These beneficial AA actions required the mTORC1–v-ATPase axis, adaptor protein Ragulator, and endosomal/lysosomal AA transporter SLC38A9, indicating an endosome-centric inside-out AA sensing mechanism. In high-fat diet-fed rats, lysine/leucine/arginine had similar beneficial actions at the myocellular level as *in vitro* in lipid-overexposed cardiomyocytes and partially reversed cardiac hypertrophy.

**Conclusion:**

Specific AAs acting through v-ATPase reassembly reduce cardiac lipid uptake raising the possibility for treatment in situations of lipid overload and associated insulin resistance.

## Highlights

• High physiological concentrations of specific AAs (K/L/R) activate v-ATPase.
• The KLR mix activates v-ATPase by mutually dependent activation of mTORC1.
• KLR-induced v-ATPase activation enables endosomes to retain lipid transporter CD36.
• KLR mends lipid-induced insulin resistance and cardiomyocytic contractile dysfunction.
• KLR reverses v-ATPase disassembly and cardiac hypertrophy in high-fat diet-fed rats.

## Graphical abstract

**Figure:**
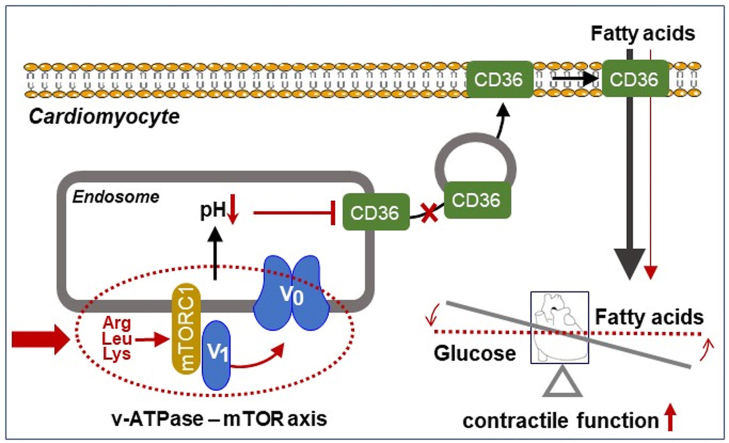

## Abbreviations

AA: amino acid
ARCMs: adult rat cardiomyocytes
BafA: Bafilomycin-A
BSA: bovine serum albumin
CHLQ: chloroquine
GLUT4: glucose transporter-4
HFD: high-fat diet
hiPSC-CMs: human-induced pluripotent stem cells differentiated into cardiomyocytes
HP: high palmitate
IP: immunoprecipitation
KLR: (mixture of) lysine, leucine, and arginine
LFD: low-fat diet
LP: low palmitate
mTORC1: mammalian target of rapamycin complex-1
v-ATPase: vacuolar-type H^+^-ATPase
V_0_: membrane-associated v-ATPase subcomplex
V_1_: cytoplasmic v-ATPase subcomplex

## 1 Introduction

Heart failure is among the most common causes of mortality in type 2 diabetic (T2D) patients [1,2] and is closely associated with several risk factors such as microvascular damage, fibrosis, oxidative stress, inflammation, and increased plasma lipid levels [3]. In rodent models of diabetes, lipids alone might lead to heart failure [4], whereas in humans, there may be an interplay between lipids and other factors. Importantly, both in rodents and humans, the diabetic heart is characterized by extensive lipid accumulation [[5], [6], [7]]. This myocellular lipid accumulation is predominantly caused by increased uptake of fatty acids by the membrane lipid transporter CD36 (SR-B2) [8].

In the healthy heart, CD36 is localized for a large part in intracellular membrane compartments, specifically the endosomes, which are characterized by luminal acidification. Upon long-term overexposure of the heart to lipids, CD36 is expelled from the endosomes and translocates to the sarcolemma. This initiates a vicious cycle of increased fatty acid uptake and lipid accumulation, ultimately culminating in cardiac contractile dysfunction [[9], [10], [11]]. Therefore, pharmacological inhibition of CD36 translocation might be a valuable treatment to counteract lipid accumulation and lipid-induced contractile dysfunction in the diabetic heart [12].

Recently, we revealed the regulatory mechanism underlying lipid-induced CD36 translocation. Excess palmitate taken up by cardiomyocytes using CD36 is intracellularly sensed by vacuolar-type H^+^-ATPase (v-ATPase) [9]. V-ATPase is commonly referred to as the endosomal/lysosomal proton pump and is responsible for acidifying the lumen of these organelles. This protein complex consists of >14 subunits divided over two subcomplexes. The membrane-inserted V_0_ subcomplex mediates the transmembrane proton movement, while the cytoplasmic V_1_ subcomplex contains an ATP hydrolysis-driven rotor enabling the pumping of protons against a gradient into the endosomes/lysosomes. Sensing of lipids (e.g., palmitate) by v-ATPase involves disassembly of V_1_ from V_0_ and disappearance of V_1_ into the cytoplasm. This results in decreased proton pump activity and the loss of endosomal acidification. The deacidified endosomes can no longer serve as a storage compartment for CD36; and hence, CD36 is forced to translocate to sarcolemma [9]. Hence, induction of v-ATPase reassembly could be an effective strategy to combat lipid-induced insulin resistance and contractile dysfunction.

In our quest for strategies to reassemble v-ATPase, we considered its dynamic participation in the formation of lysosome-associated protein super-complexes, which recently received considerable attention. For example, v-ATPase is implicated as an essential component in the docking of the anabolic master regulator mTORC1 to the lysosomal membranes and its subsequent activation by amino acids (AAs) [13,14]. Specifically, AAs induce mTORC1 activation in accordance with an endosomal/lysosomal-centric inside-out sensing mechanism [13]. This mechanism entails that AAs first accumulate inside lysosomes, and subsequently, move out of the lysosomes through the lysosomal AA transporter SLC38A9. It is this lysosomal AA efflux that is sensed by mTORC1, but only when mTORC1 is bound to v-ATPase through the adaptor protein complex Ragulator.

In the present study, we speculated that the relationship between v-ATPase and mTORC1 can work both ways, i.e., the AA-induced interaction between v-ATPase and mTORC1 not only does activate mTORC1, but also mutually activates v-ATPase. Hence, we hypothesized that by the addition of AAs, v-ATPase can be activated so that endosomes are reacidified in an mTORC1-dependent manner, thereby allowing the reinternalization of CD36, which would limit excessive lipid uptake and resolve lipid-induced insulin resistance and contractile dysfunction. To investigate this hypothesis, we first set out to identify individual AAs on their ability to activate v-ATPase and to uncover the underlying mechanism. Subsequently, the three most effective AAs (lysine/leucine/arginine) were combined into a cocktail for studies *in vitro* in lipid-overexposed cardiomyocytes and *in vivo* in hearts from rats fed with a high-fat diet (HFD). We conclude that specific AA supplementation antagonizes lipid-induced contractile dysfunction through mutual mTORC1–v-ATPase activation.

## 2 Methods

### 2.1 Reagents and antibodies

Detailed information on reagents, antibodies, and primers is provided in Supplementary Tables S1–2.

### 2.2 Animal care and use

Male Lewis rats (250–300 g) were purchased from Charles River laboratories and maintained at the Experimental Animal Facility of Maastricht University. Animals were housed in a controlled environment (21–22 °C) on 12:12 h light–dark cycle, and had free access to food and tap water. All animal experiments were performed according to Dutch regulations and approved by the Dutch Central Committee of Animal Use (CCD) and the Maastricht University Committee for Animal Welfare.

### 2.3 Culturing of adult rat cardiomyocytes (aRCMs), HL-1 cardiomyocytes, and human-induced pluripotent stem cells differentiated into cardiomyocytes (hiPSC-CM)

Three different cardiomyocyte models were employed in the course of this study. Each of these models has its merits and disadvantages. HL-1 cells have the advantage of easy transfection, but have a lower level of lipid metabolism and CD36 expression compared to primary cardiomyocytes. However, primary rat cardiomyocytes are extremely difficult to transfect. Yet, these primary cardiomyocyte cultures retain the ability to contract (when subjected to electric field stimulation). Finally, the hiPSC-CMs are important to extend the findings concerning the beneficial metabolic effects of the KLR mix to the human setting. However, human stem cells culture is time-consuming that involves complex maturation protocols.

HL-1 cells were cultured as previously described [15]. HiPSC-CM were generated and cultured as described recently [16]. To investigate whether AAs can alter v-ATPase activity, HL-1 cells were first subjected to complete AA starvation for 1 h followed by control medium (DMEM-F12) without/with 4∗ individual AA readdition for 1 h. To investigate potential protective actions of the 4∗KLR cocktail during excess lipid supply, HL-1 cells and hiPSC-CM were cultured for 24 h in either control medium (no palmitate) or HP medium (500 μM palmitate; palmitate/BSA ratio 6:1), and also with HP/4∗KLR medium as previously described [9].

Adult rat cardiomyocytes (aRCMs) were isolated using a Langendorff perfusion system [9,17]. Cells were used for subsequent studies, provided that >80% of the cells were rod-shaped and excluded trypan blue [17]. After 2 h of adhesion, aRCMs were cultured for 24 h in either low-palmitate medium (LP; 20 μM palmitate; palmitate/BSA ratio 0.3:1), high-palmitate medium (HP; 200 μM palmitate; palmitate/BSA ratio 3:1), or HP medium supplemented with lysine/leucine/arginine at 1.36/1.84/1.56 mM, representing 4x their low-physiological plasma (HP/4∗KLR). For further details, refer [16]. It should be noted that in contrast to HL1 cells and hiPSC-CMs – where a zero-palmitate containing control condition is used to evaluate the effects of HP culturing – in case of aRCM, the LP condition serves as the insulin-sensitive control condition for comparison with the insulin resistance-inducing HP condition. The underlying reason is that the aRCM does not survive culturing in the absence of fatty acids.

### 2.4 Transfection with siRNA

HL-1 cells (70% confluency) were transfected with scrambled siRNA (50 pmol), siRNA targeting v-ATPase B2 subunit RNA (B2-kd) (50 pmol), SLC38A9 (75 pmol), or Lamtor1 (Ragulator subunit; 50 pmol) using Lipofectamine-RNAiMAX (Invitrogen, Carlsbad, CA) according to the manufacturer's protocols. After transfection, cells were kept for 24 h in a growth medium (Claycomb medium with FBS) and then exposed to depletion medium (DMEM-31885 without FBS) for 24 h. Transfection efficiency was evaluated by western blot analysis (Fig. S3).

### 2.5 Animal diets and KLR supplementation

Lewis rats (195–217 g) were randomly divided into three groups of seven animals: (1) rats were fed with a 10% low-fat diet (LFD; D12450B, Research Diet Services-BV, Wijk-bij-Duurstede, Netherlands) for 12 weeks, (2) a 60% high-fat diet (HFD; D12492) for 12 weeks, and (3) HFD for 12 weeks – with the last 4 weeks on drinking water containing H-KLR cocktail (Lys/Leu/Arg: 7/12/10 mM).

### 2.6 Echocardiography

Noninvasive trans-thoracic echocardiograms were recorded using a Vevo-2100 High-Resolution Imaging system with a nonlinear MS250 Transducer (13–24 MHz, VisualSonics, Toronto, Canada). Rats were induced with 3–4% isoflurane and maintained with 1.5% isoflurane. Serial echocardiography was performed at 0/4/8/10/12 weeks after start diet, with analysis blinded to study groups.

### 2.7 Plasma and tissue determinations

Blood glucose was measured using an ACCU-CHEK Performa Glucometer (Roche Diagnostics, Mannheim, Germany). Insulin levels were estimated in plasma samples using a rat insulin enzyme-linked immunosorbent assay (ELISA) kit (Sigma–Aldrich., St. Louis). Plasma levels of lipids were determined with a Liquicolor Triglyceride determination kit (Sigma–Aldrich., St. Louis). After euthanization, hearts were snap-frozen in liquid nitrogen and stored at −80 °C.

### 2.8 Amino acid (AA) measurements

Blood samples were collected into EDTA-containing tubes and deproteinated as previously described [18]. Plasma AA concentrations were determined by HPLC after precolumn derivatization with o-phthaldialdehyde [18].

### 2.9 Western blotting of lysates from heart

Rat heart tissue samples were homogenized and subsequently used for western blotting as previously described [9].

### 2.10 Measurement of v-ATPase disassembly/assembly

As previously described [9], two methods were applied to measure disassembly: immunoprecipitation (IP) and subcellular fractionation. Immunoprecipitates and membrane/cytoplasmic fractions were used for western detection of v-ATPase-a2 and d1 (subunits of membrane-bound V_0_), and also B2 (subunit of cytoplasmic V_1_).

### 2.11 Measurement of cellular chloroquine (CHLQ) accumulation as a readout of v-ATPase function

[^3^H]CHLQ accumulation in cultured cells was measured as previously described [9].

### 2.12 Surface-protein biotinylation for detecting GLUT4 and CD36 translocation

Surface-protein biotinylation was measured as previously described [19]. Briefly, after a 24 h culture under various conditions, aRCMs were incubated for 30 min with (or without) 100 nM insulin. During the last 10 min of this period, the cell-impermeable reagent sulfo–NHS–LC-biotin (Thermo Fisher Scientific, Fremont, CA) was added. Thereafter, the cells were washed and lysed for subsequent IP with streptavidin beads (Thermo Fisher Scientific). Upon further washing and elution of the biotinylated proteins from the beads, samples that contained the biotinylated proteins were used for the western analysis of CD36 and insulin-regulated aminopeptidase (IRAP, reflecting GLUT4 trafficking).

### 2.13 Quantification of triacylglycerol contents

Triacylglycerol contents were performed using a Triglyceride Assay Kit (ab65336, Abcam, San Francisco, CA) following the manufacturer's instructions. Triacylglycerol was normalized to protein concentration.

### 2.14 Determination of insulin signaling

Insulin signaling was measured as previously described [16]. Briefly after culturing, cells were exposed to 100 nM insulin for 30 min, followed by lysis and western blot analysis of p-mTOR (ser2448), p-AKT (ser473), total-AKT, p-S6 ribosomal protein (ser235/236), and caveolin-3 (Cav-3).

### 2.15 Measurement of substrate uptake

[^14^C]Palmitate and [^3^H]deoxyglucose uptake rates into cardiomyocyte cultures were measured as previously described [9].

### 2.16 Measurement of cardiomyocyte contraction dynamics

Contractile properties of aRCMs were measured using a video-based cell geometry system (IonOptix, Milton, MA). Cells were electrically paced in Tyrode buffer at 20-Volts, 1-Hz, and 37 °C. Line-scan images of 10–15 successive beats were recorded with IonWizard acquisition software for the calculation of contractile parameters [9].

### 2.17 Evaluation of GLUT4 translocation

For the evaluation of GLUT4 translocation, aRCMs were transduced with an adenoviral construct containing HA-GLUT4-GFP [20]. The HA-tag was used for immunostaining and microscopical imaging of GLUT4. Quantification relative to GFP was performed by ImageJ.

### 2.18 Statistics

Statistical analyses were performed using IBM SPSS Statistics 23 (SPSS Inc., Chicago, IL) and GraphPad 8.0 PRISM®. Briefly, *in vitro* data were compared using a one-way ANOVA followed by Duncan's post hoc tests (among the groups, i.e., different culturing conditions), or paired Student's t-test (within groups, i.e., when analyzing short-term insulin effect). Additionally, *in vivo* data were compared using a one-way ANOVA followed by Duncan's post hoc tests (among groups). All data are presented as mean ± SEM. P-values <0.05 are considered statistically significant.

## 3 Results

### 3.1 Effects of AA treatment on v-ATPase disassembly and deactivation in lipid-overexposed cardiomyocytes

**Figure 1:**
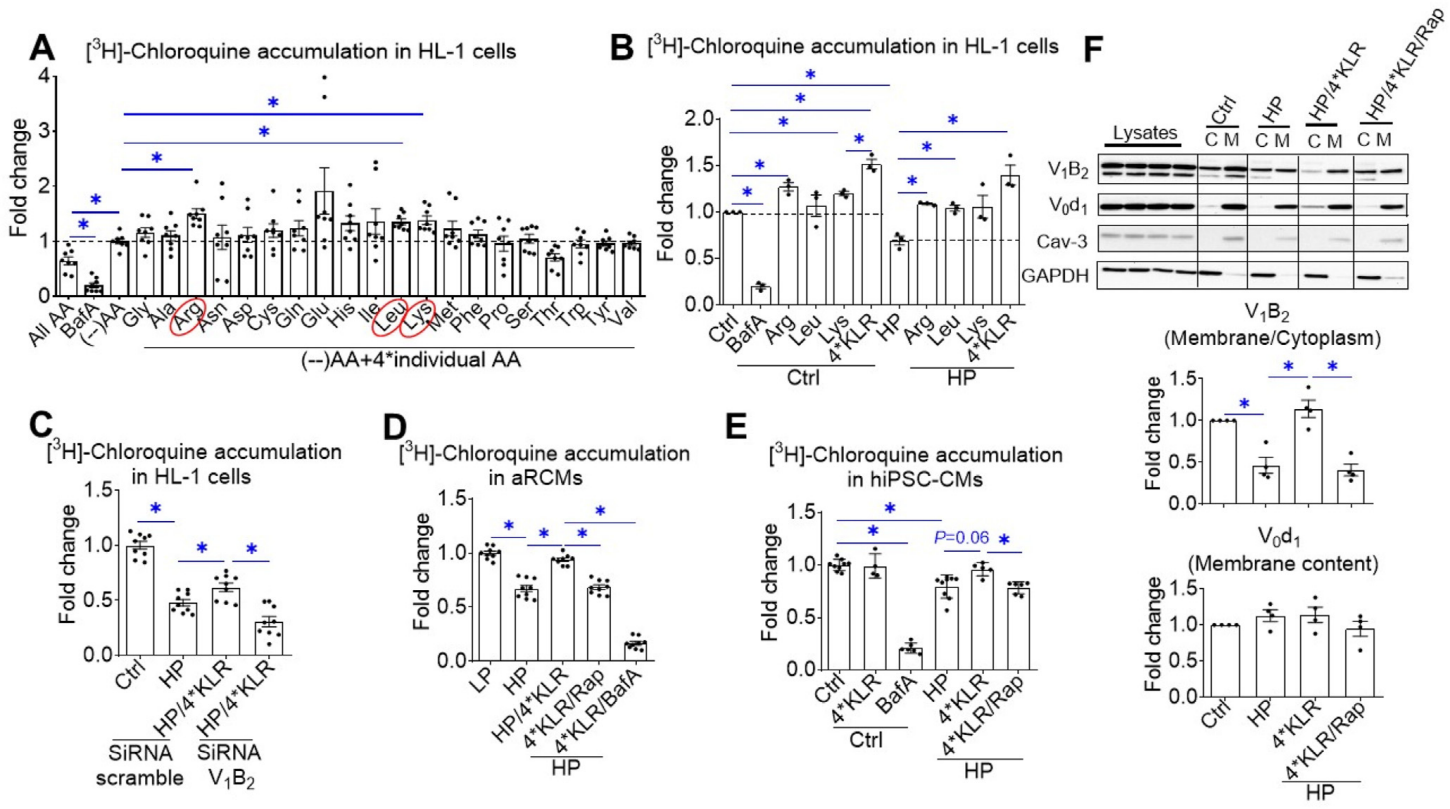
**Amino acids (AA) treatment prevents v-ATPase disassembly and deactivation in lipid-overexposed cardiomyocytes**. **A.** Effects of individual AAs on [^3^H]Chloroquine (CHLQ) accumulation in HL-1 cardiomyocytes. Cells were first subjected to complete AA starvation for 1 h followed by further culturing for 1 h in either control medium ((--)AAs), control medium with all AAs at 1∗ concentration (All AAs), control medium with 100 nM v-ATPase inhibitor Bafilomycin A, or re-addition of each of the individual AAs (at 4∗concentrations) to control medium. Then, cells were subjected to the [^3^H]CHLQ accumulation assay. Data are means ± SEM. n = 6. **B–F.** Cells were cultured for 24 h under various conditions, being control (Ctrl: no palmitate; the basal condition for HL-1 and hiPSC-CMs), low palmitate (LP, basal condition for aRCMs; palmitate/BSA ratio 0.3:1), high palmitate (HP, palmitate/BSA ratio 3:1), LP or HP supplemented with 4∗KLR cocktail (HP/4∗KLR), HP/4∗KLR supplemented with 100 nM rapamycin (HP/4∗KLR/Rap) or 100 nM BafA (HP/4∗KLR/BafA). **B-E.** Cell-associated [^3^H]CHLQ accumulation**. B–C.** Hl-1 cells. **B.** Comparison of the effects of 4∗KLR mixed with that of the individual AA components at 4∗ concentrations (n = 3). **C.** HL-1 was transfected with scrambled siRNA or with siRNA targeting the v-ATPase B2 subunit. 32 h after transfection, cells were cultured under the various mentioned conditions (n = 9). **D.** aRCMs. n = 4. **E.** hiPSC-CMs. n = 6. **F.** Subcellular fractionation of HL-1 cells. Cytoplasmic fractions (C) and membrane fractions (M) were analyzed by Western blotting of v-ATPase subunits B2 (V_1_–B2) and d1 (V_0_-d1), after which the signals were quantified. For V_1_–B2, the signal ratio of membrane fraction/cytoplasm in the control condition is set at 1.0. For V_0_-d1, the signal density in the membrane fraction is 1.0. The quantified signals in the other conditions are expressed as multiples. Representative blots are displayed. Caveolin-3 (Cav-3) and GAPDH: loadings controls for membrane and cytoplasmic fraction, respectively. n = 4. Bar values are means ± SEM. ∗*p* < 0.05.

To select the most potent AAs for v-ATPase activation, we tested each AA on its ability to increase the cell-associated accumulation of the divalent weak base chloroquine (CHLQ) in a radioactivity-based assay. CHLQ becomes specifically trapped in acidic organelles, such as endosomes, and when added to cells in trace amounts, it provides quantitative information about luminal acidification [21]. For defining cell-associated CHLQ accumulation under baseline conditions, HL-1 cardiomyocytes were incubated in a control medium with no AAs (Figure 1A). Approximately 80% of this CHLQ amount is inhibited by the incubation of cells with the v-ATPase inhibitor Bafilomycin A (BafA), and thus, owing to v-ATPase activity, the remainder likely represents cellular acidification processes independent of v-ATPase. When a mixture of all AAs was added at similar (1x) concentrations of each AA as occurring in DMEM-F12 (reflecting their presence in the circulation of 24 h-starved rats) [22],v-ATPase activity was partly inhibited (Figure 1A) in agreement with earlier work in HEK293T cells [23]. When each AA was added individually at 4x its low physiological concentration, several AAs appeared to stimulate v-ATPase activity to some extent in HL-1 cells, but only lysine (/(K), leucine (/L), and arginine (/R) exerted statistically significant effects (Figure 1A), The stimulatory action of these three AAs was confirmed in HEK293-cells (Supplementary Fig. S1). For subsequent experiments, these three AA species were added in a combined manner at their 4x concentration (designated 4∗KLR; Supplementary Table S3).

To test the 4∗KLR cocktail, three cardiac cell models were employed: next to HL-1cells, also aRCM and hiPSC-CM (for characteristics refer Supplementary Fig. S2). In HL-1 cells, v-ATPase activity decreased in HP media culture (Figure 1B–C), as previously observed [9]. The 4∗ KLR cocktail increased v-ATPase activity both under control culture and on HP exposure. Additionally, the 4∗KLR-mediated increase in v-ATPase activity exceeded the increases exerted by the individual AA in this cocktail (Figure 1B). Just as in HL-1 cells, HP exposure resulted in a decrease in v-ATPase activity in aRCM and hiPSC-CM (Figure 1D–E), and when HP exposure was combined with the 4∗KLR cocktail, v-ATPase activity was completely preserved (at least in HL-1 cells, with a tendency for preservation in hiPSC-CM; P = 0.06; Figure 1D–E). SiRNA-mediated silencing of the v-ATPase-B2 subunit or BafA-mediated inhibition of v-ATPase abolished this AA-action (Figure 1C–D; Supplementary Fig. S3A). This effect was also sensitive to the specific mTOR inhibitor rapamycin (Figure 1D–E), indicating that mTORC1 activation is necessary for KLR-induced v-ATPase activation during lipid overload, and that this AA action is evolutionarily conserved from rodents to humans. Rapamycin also inhibits v-ATPase activity in cardiomyocytes under control conditions (to a modest extent; Supplementary Fig. S4A), indicating that mTOR also plays a role in basal v-ATPase-mediated proton pumping in accordance with basal phosphorylation of mTORC1 at ser2448 (Supplementary Fig. S5A).

Lipid-induced v-ATPase inhibition is because of v-ATPase disassembly into its two subcomplexes as recently observed [9]. Using subcellular fractionation of HL-1 cells, we confirmed that in all conditions, the V_0_-d1 subunit (within the membrane-bound V_0_ subcomplex) was retained in the membrane fraction (Figure 1F). On HP exposure, the V_1_–B_2_ subunit (within the soluble V_1_ subcomplex) relocalized from the membrane fraction to the cytoplasmic fraction (Figure 1F), indicating V_0_/V_1_ disassembly. Notably, when HP-exposed cardiomyocyte cultures were simultaneously treated with 4∗KLR, V_1_–B2 was redistributed back to the membrane fraction in a rapamycin-sensitive manner (Figure 1F). In accordance with the data from the CHLQ assay, treatment with 4∗KLR through mTORC1 activation preserves v-ATPase assembly; and thus, v-ATPase activity and endosomal acidification are maintained during lipid overload.

### 3.2 Mechanism of AA sensing involved in v-ATPase reassembly in lipid-overloaded cardiomyocytes

**Figure 2:**
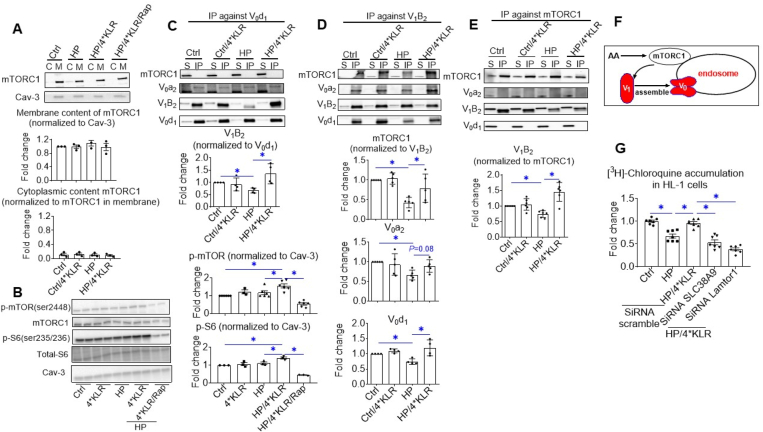
**Mechanism of AA sensing involved in v-ATPase reassembly in lipid-overloaded cardiomyocytes**. In all panels, cells were cultured for 24 h under various conditions, being Ctrl (no palmitate; the basal condition for HL-1 and hiPSC-CMs), low palmitate (LP, basal condition for aRCMs), high palmitate (HP, palmitate/BSA ratio 3:1), HP supplemented with 4∗KLR (HP/4∗KLR), or HP/4∗KLR supplemented with 100 nM Rap (HP/4∗KLR/Rap). **A.** Subcellular localization of mTORC1. Upon subcellular fractionation of HL-1 cells, Cytoplasmic fractions (C) and membrane fractions (M) were analyzed by Western blotting of (total) mTORC1, after which the signals were quantified. A representative blot is displayed Caveolin-3 (Cav-3): loading control. n = 3. **B.** Activation of mTORC1. Phosphorylation and expression of mTORC1 (p-mTOR ser2448 and total mTORC1) and of S6 (p-S6 ser235/236 and total S6) were detected by Western blotting (n = 3–6). Representative blots are displayed. For quantitative comparison of p-mTOR and p-S6 among the different conditions, these signals were normalized against the respective signal of caveolin-3 protein content (loading control). **C–E.** Co-Immunoprecipitation (Co-IP) of V_1_, V_0_, and mTORC1 in HL-1 cells. **C.** IP with V_0_-d1. **C.** IP with V_1_–B2. **E.** IP with mTORC1. Immunoprecipitates were blotted with antibodies against mTORC1, V_0_-a2, V_0_-d1, and V_1_–B2, after which the signals were quantified. Representative western blots are displayed. n = 5. **F.** Scheme illustrating how AAs preserve v-ATPase assembly in lipid-overexposed cardiomyocytes involving mTOR activation. **G.** Cell-associated [^3^H]CHLQ accumulation upon silencing of SLC38A9 or Lamtor-1. HL-1 was transfected with scrambled siRNA or with siRNA targeting SLC38A9 or Lamtor-1. After 32 h transfection, cells were cultured under the following conditions: Ctrl, HP, or HP/4∗KLR. n = 7. Bar values are means ± SEM. ∗*p* < 0.05.

We first examined the effect of 4∗KLR treatment on subcellular localization of mTORC1 and whether this treatment was able to activate mTORC1. Already under control conditions, mTORC1 was entirely associated with membranes with no alterations under HP condition and/or 4∗KLR treatment (Figure 2A). The 4∗KLR mixture stimulated phosphorylation of mTORC1-ser2448 and downstream S6-ser235/236 in HL-1 cells (both by ~1.5-fold); but only in the HP condition, and not in the control condition (Figure 2B; Supplementary Fig. S5A). Possibly, mTOR activation by AA requires some minimal amount of palmitate or other fatty acids, which are absent in the control culturing condition. In accordance with this, mTORC1 signaling is palmitoylation-dependent in mammalian cells [24]. Importantly, the 4∗KLR-mixture activates mTORC1 in lipid-overloaded cardiomyocytes. However, this activation does not include mTORC1 migration from the cytoplasm to (endosomal) membranes as mTORC1 is already membrane-bound under baseline conditions (Figure 2A).

Next, we investigated whether this AA-induced mTORC1 activation in HP-exposed cells was associated with mTORC1 binding to v-ATPase, and would accompany KLR-induced v-ATPase reassembly. Besides, by using fractionation (Figure 1F), the v-ATPase assembly state can be measured through immunoprecipitation (IP). This requires assessment of the degree of co-IP of subunits that reside in each of the two different subcomplexes by using antibodies that recognize these specific subunits. For this, we checked the presence of the V_1_-subunit B2 in an IP against the V_0_-subunit d1 (Figure 2C) and the presence of the V_0_-subunits a2 and d1 in an IP against B2 (Figure 2D). These IPs confirm the findings of the fractionation experiment: HP exposure induces v-ATPase disassembly, which is prevented by 4∗KLR treatment. These IPs were also assessed on the presence of mTORC1. In the LP condition when v-ATPase is assembled, mTORC1 binds only to the V_1_-subcomplex, and not to V_0_ (Figure 2C–D). These findings were confirmed in the reverse IP against mTORC1 – wherein the LP condition, the B2 subunit, but not a2 and d1 were detected (Figure 2E). HP exposure decreased mTORC1 binding to V_1_, which was prevented by 4∗KLR treatment (Figure 2C–E). Taken together in lipid-overexposed cells, 4∗KLR treatment results in mTORC1 activation, which attracts the V_1_-_-_subunit to the endosomal membrane, allowing V_1_ to reassemble with V_0_ (Figure 2F). Notably, silencing of SLC38A9 or Lamtor1 (Ragulator-subunit) proteins abrogated the AA-induced v-ATPase reactivation (Figure 2G; Supplementary Figs. S3B–C), indicating that 4∗KLR treatment exerts this beneficial action by the inside-out mechanism of endosome/lysosome-centric AA sensing and involving Ragulator for mTORC1 binding to v-ATPase.

### 3.3 Effects of AA treatment on CD36-mediated lipid accumulation in lipid-overexposed cardiomyocytes

**Figure 3:**
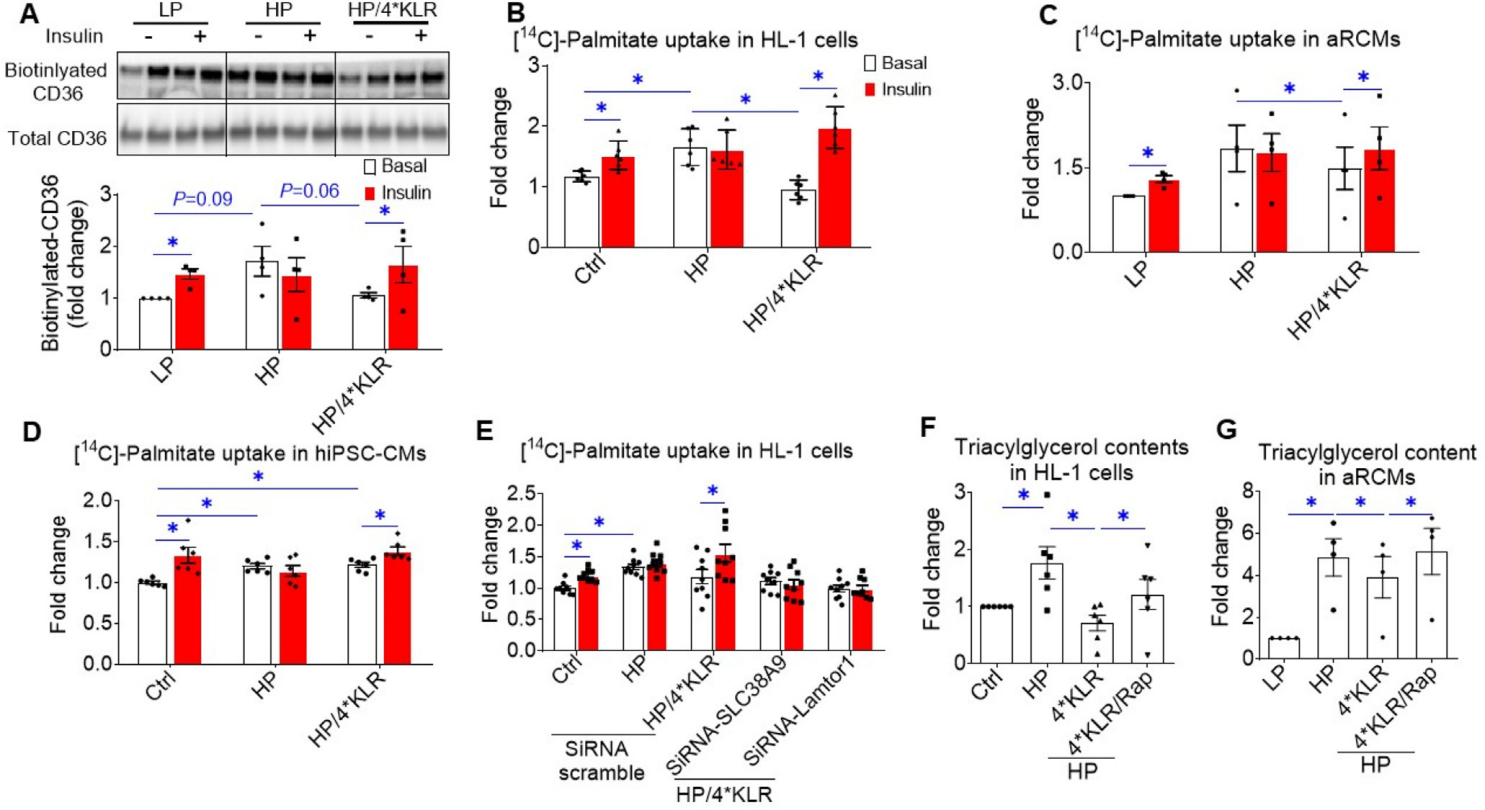
4**∗KLR treatment prevents CD36-mediated lipid accumulation in lipid-overexposed cardiomyocytes**. In all panels, cells were cultured for 24 h under various conditions, being Ctrl (no palmitate; the basal condition for HL-1 and hiPSC-CMs), low palmitate (LP, basal condition for aRCMs), high palmitate (HP, palmitate/BSA ratio 3:1), HP supplemented with 4∗KLR (HP/4∗KLR), HP/4∗KLR supplemented with 100 nM Rap (HP/4∗KLR/Rap). **A–E.** After 24 h, cells were short-term (30 min) incubated without/with insulin (HL-1 cells and hiPSC-CMs: 200 nM insulin; aRCMs: 100 nM insulin). **A**. Assessment of cell-surface CD36 in aRCMs using biotinylation assay. For this, CD36 was detected by Western blotting in biotin-immunoprecipitated and total lysate fractions, and subsequently, quantified. n = 4. **B–D**. [^14^C]palmitate uptake in HL-1 cells (n = 6), aRCM (n = 3) and hiPSC-CMs (n = 6). **E**. [^14^C]palmitate uptake in HL-1 cells upon silencing of SLC38A9 or Lamtor-1. HL-1 was transfected with scrambled siRNA or with siRNA targeting SLC38A9 or Lamtor-1. Upon 32 h after transfection, cells were cultured under the following conditions: Ctrl, HP, or HP/4∗KLR (n = 9). **F-G**. Triacylglycerol contents in HL-1 cells (n = 4) and aRCM (n = 4). Bar values are means ± SEM. ∗*p* < 0.05.

In lipid-overloaded cardiomyocytes, v-ATPase inhibition leads to increased CD36 translocation to the sarcolemma and myocellular lipid accumulation [9]. Using a surface biotinylation assay, we confirmed the HP-induced CD36 translocation (1.5-fold increase in basal cell-surface CD36; Figure 3A; P = 0.08; Supplementary Fig. S4B: P = 0.02), which was not altered by rapamycin (Supplementary Fig. S4B). Insulin did not further stimulate CD36 translocation in the HP condition (Figure 3A), confirming that lipid-overload induces CD36 translocation from insulin-responsive endosomal stores [9]. The 4∗KLR cocktail tended to prevent the HP-induced increase in basal CD36 translocation (P = 0.06), and also completely preserved insulin-stimulated CD36 translocation (Figure 3A). When fatty acid uptake was studied, similar trends were observed in all three cardiomyocyte models: an HP-induced increase with loss of insulin stimulation, which was reversed by the 4∗KLR cocktail (Figure 3B–D). Coupling the 4∗KLR treatment to simultaneous silencing of SLC38A9 or Lamtor1 rendered the 4∗KLR cocktail unable to preserve insulin-stimulated fatty acid uptake (Figure 3E), indicating that AA-induced preservation of insulin-stimulated fatty acid uptake relies on AA-sensing by mTORC1 through the endosome/lysosome-centric mechanism and on mTORC1 binding to v-ATPase.

The AA-induced decrease in CD36 translocation and fatty acid uptake in lipid-overexposed cardiomyocytes is expected to impact myocellular lipid accumulation. However, myocellular triacylglycerol content was 2–4-fold increased in cardiomyocytes upon HP culturing (Figure 3F–G). In addition, the 4∗KLR mixture largely prevented this increase, while this effect was lost in the presence of rapamycin (Figure 3F–G). Altogether, in lipid-exposed cardiomyocytes, 4∗KLR treatment prevents CD36-mediated lipid accumulation in an mTORC1-dependent manner.

### 3.4 Effects of AA treatment on the loss of insulin signaling and loss of insulin-stimulated glucose uptake in lipid-overexposed cardiomyocytes

**Figure 4:**
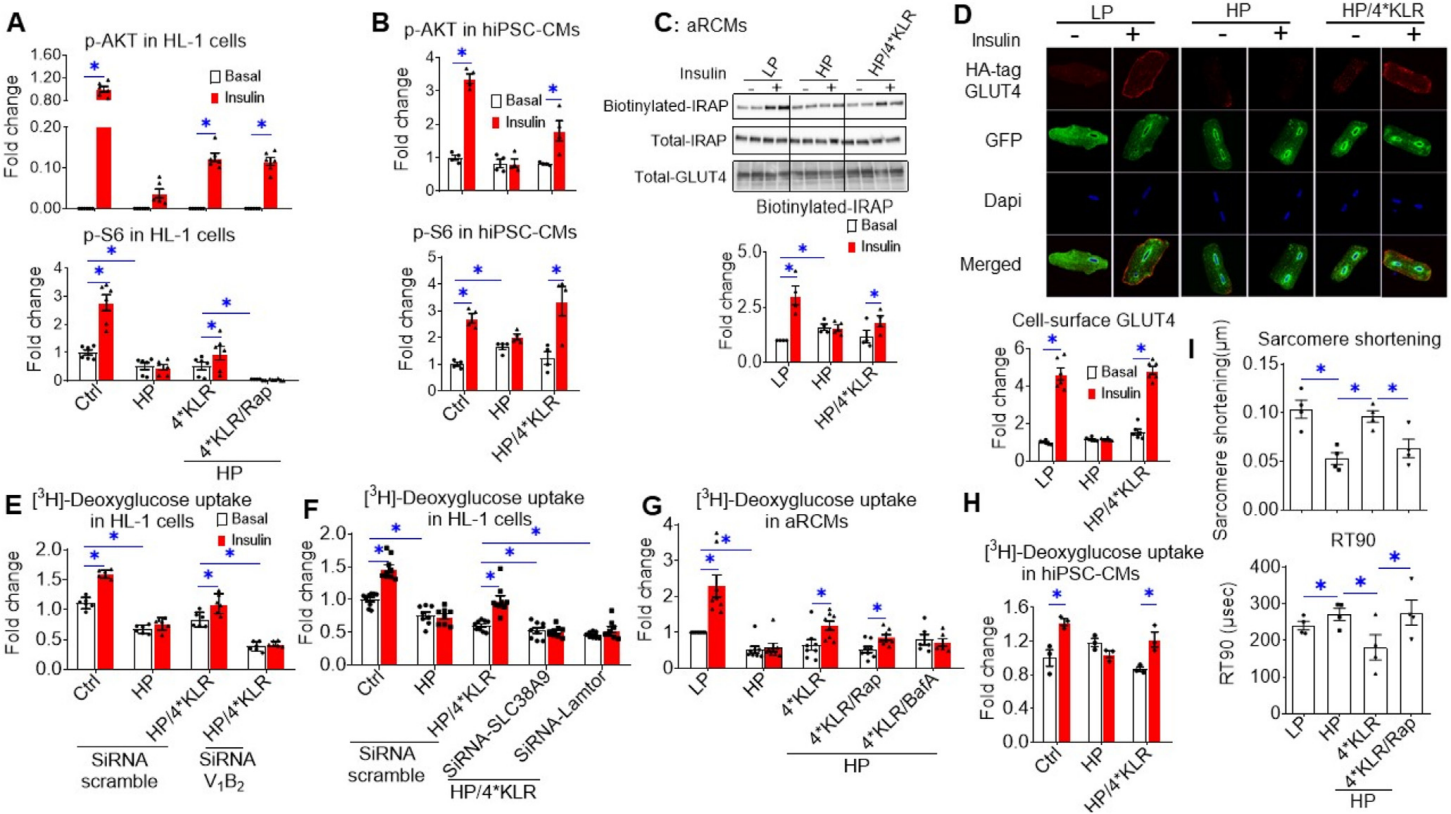
**4∗KLR treatment partially preserves insulin signaling in lipid-overexposed cardiomyocytes, but completely preserves insulin-stimulated glucose uptake and contractile activity.** In all panels, cells were cultured for 24 h under various conditions, being Ctrl (no palmitate; the basal condition for HL-1 and hiPSC-CMs), low palmitate (LP, basal condition for aRCMs), high palmitate (HP, palmitate/BSA ratio 3:1), HP supplemented with 4∗KLR (HP/4∗KLR), HP/4∗KLR supplemented with 100 nM Rap (HP/4∗KLR/Rap) or 100 nM BafA (HP/4∗KLR/BafA). **A–H.** After 24 h, cells were short-term (30 min) incubated without/with insulin (HL-1 cells/hiPSC-CMs: 200 nM insulin; aRCMs: 100 nM insulin). **A–B.** Phosphorylation of AKT (p-AKT ser473) and of S6 (p-S6 ser235/236) in HL-1 cells (n = 6) and hiPSC-CMs (n = 3) were detected by western blotting. For quantitative comparison of p-AKT and p-S6 among the different conditions, these signals were normalized against the respective signal of caveolin-3 protein content (loading control). Representative western blots of p-AKT and p-S6, and also of total AKT and Cav-3 are displayed in Supplementary Figs. S5B–C. **C–D**. Assessment of the cell surface GLUT4 in aRCMs. **C.** Biotinylation assay. Representative western blot and quantification of insulin-regulated aminopeptidase (IRAP, which reflects GLUT4 translocation) in biotin-immunoprecipitation and total lysate fraction. n = 4. **D.** Microscopical assay. aRCMs were infected for 48 h with an adenoviral vector containing HA-GLUT4-GFP fusion protein, and the last 24 h included the culturing under LP, HP, and HP/4∗KLR conditions, as described above. After this 48 h period, cells were subjected to short-term insulin stimulation. Nonpermeabilized cells were anti-HA immunostained. The nuclei are stained in blue (DAPI). Representative microscopical images are displayed. The ratios of red (HA-tag) and green (GFP) intensity per pixel were quantified by Image J. n = 3. **E–H.** [^3^H]Deoxyglucose uptake in HL-1 cells (n = 8), aRCMs (n = 8) and hiPSC-CMs (n = 3). **E–F.** [^3^H] Deoxyglucose uptake in HL-1 cells upon silencing of V_1_B2, SLC38A9, or Lamtor-1. HL-1 was transfected with scrambled siRNA or with siRNA targeting V_1_B2, SLC38A9, or Lamtor-1. Upon 32 h after transfection, cells were cultured under the following conditions: Ctrl, HP, or HP/4∗KLR. n = 8. **I.** Contractile parameters of aRCMs. Sarcomere shortening and decay time to 90% of its peak (RT90) were measured by video imaging of aRCMs during electrostimulation at 1 Hz frequency. Bar values are means ± SEM. ∗*p* < 0.05.

Increased CD36-mediated fatty acid uptake and lipid accumulation precede the development of insulin resistance [25]. For evaluation of insulin signaling, p-AKT (ser473) and downstream p-mTOR (ser2448) and p-S6 (ser235/236) were assessed. As expected [9], HP exposure caused a loss of insulin-stimulated phosphorylation of these proteins. The 4∗KLR cocktail partially prevented this loss of insulin signaling (Figure 4A–B; Supplementary Figs. S5A–C). Also as expected, rapamycin blocked the AA-induced rephosphorylation of mTOR and its downstream target S6, but not of its upstream effector AKT (Figure 4A–B; Supplementary Figs. S5A–C).

Next, a surface detection assay of insulin-responsive aminopeptidase (IRAP) was applied to study GLUT4 translocation. Insulin-stimulated GLUT4 translocation was abolished upon HP exposure, but was partly preserved when HP exposure was combined with 4∗KLR addition (Figure 4C). Microscopic inspection of cell surface levels of HA-tagged GLUT4 in adenovirally transfected aRCM yielded the same results: 4∗KLR cocktail protects against HP-induced loss of insulin-stimulated GLUT4 translocation (Figure 4D). We also measured myocellular glucose uptake. As expected [9,16], HP exposure of cardiomyocytes led to the loss of insulin-stimulated glucose uptake, which was again rescued by the 4∗KLR cocktail (Figure 4E–H). SiRNA silencing of v-ATPase-B2, SLC38A9, or Lamtor1, or treatment with BafA or rapamycin (Figure 4E–G) abrogated this beneficial AA action; rendering the mTORC1-v-ATPase axis necessary for 4∗KLR-mediated preservation of insulin sensitivity in lipid-overexposed cardiomyocytes.

### 3.5 Effect of AA treatment on contractile dysfunction in lipid-overexposed cardiomyocytes

Culturing of aRCM in HP media results in contractile dysfunction [26], which was confirmed in the present study (50% reduction in sarcomere shortening and a modest increase in decay time; Figure 4I). This contractile dysfunction was not caused by cell death (Supplementary Fig. S6A). The 4∗KLR treatment prevented the negative effects of HP exposure on sarcomere shortening and other contractile parameters, while upon rapamycin addition, the AA-induced protection was lost (Figure 4I; Supplementary Fig. S6B). Hence, the 4∗KLR cocktail preserves contractile function in lipid-overloaded cardiomyocytes in an mTORC1-dependent manner.

### 3.6 Extension of AA treatment from preservation to the restoration of insulin signaling and insulin-stimulated substrate uptake in lipid-overexposed cardiomyocytes

**Figure 5:**
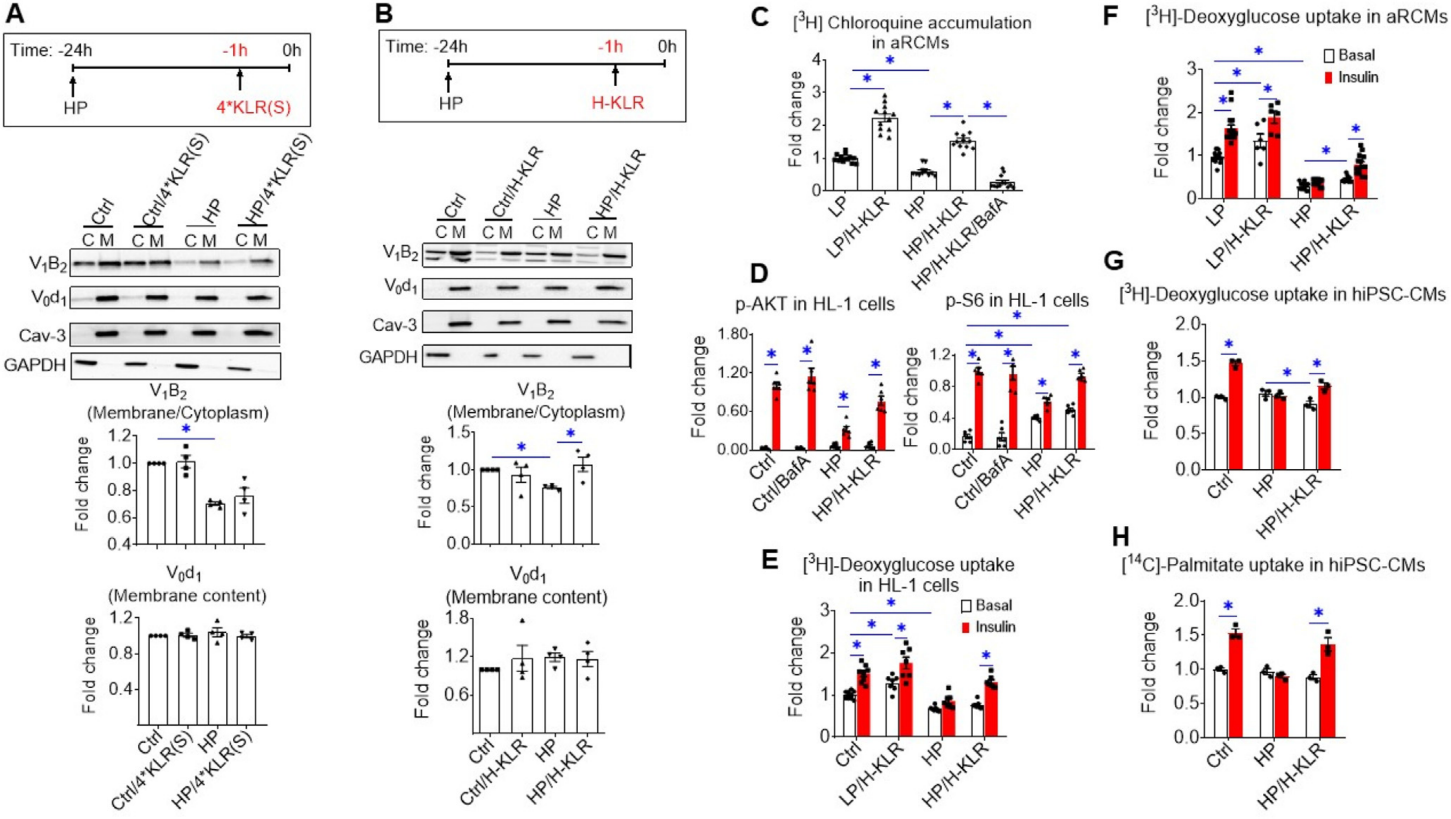
**A cocktail of Lys, Leu, and Arg not only preserves, but also restores v-ATPase assembly and insulin sensitivity in lipid-overexposed cardiomyocytes**. **A.** Effect of short-term (1 h) treatment of lipid-overexposed cardiomyocytes with 4∗KLR cocktail on v-ATPase assembly. **B–H.** Effect of short-term (1 h) treatment of lipid-overexposed cardiomyocytes with a cocktail of Lys, Leu, and Arg at >20∗ higher concentrations than in the 4∗KLR cocktail (named H-KLR) on v-ATPase assembly and insulin sensitivity. **A-B.** Subcellular fractionation of HL-1 cells. Before fractionation, HL-1 cells were cultured for 24 h with either Ctrl medium (no palmitate) or HP medium, followed by (−/+) **A.** 4∗KLR addition for 1 h (4∗KLR(S (hort)); n = 4) or **B.** H-KLR addition for 1 h (n = 4). Cytoplasmic fractions (C) and membrane fractions (M) were analyzed by western blotting of V_0_d1 and V_1_B2, after which the signals were quantified. For V_1_–B2, the signal ratio of membrane fraction/cytoplasm in the control condition is set at 1.0. For V_0_-d1, the signal density in the membrane fraction is 1.0. The quantified signals in the other conditions are expressed as multiples. A representative blot is on display. Caveolin-3 (Cav-3) and GAPDH: loadings controls for membrane and cytoplasmic fraction, respectively. **B–H.** Cells were cultured for 24 h with either Ctrl medium or HP medium, followed by (−/+) the addition (1 h) of H-KLR (HP/H-KLR) or 100 nM BafA (HP/H-KLR/BafA). **C.** Cell-associated [^3^H]CHLQ accumulation in aRCMs. n = 12. **D–H.** After 23 h culturing and subsequent 1 h H-KLR treatment, cells were short-term (30 min) incubated without/with insulin (HL-1 cells/hiPSC-CMs: 200 nM insulin; aRCMs: 100 nM insulin). **D.** Insulin sensitivity in HL-1 cells. Phosphorylation of AKT (p-AKT ser473) and of S6 (p-S6 ser235/236) in HL-1 cells (n = 6) were detected by western blotting. For quantitative comparison of p-AKT and p-S6 among the different conditions, these signals were normalized against the respective signal of caveolin-3 protein content (loading control). Representative blots of p-AKT and p-S6, and also of total AKT and Cav-3 are displayed in Supplementary Fig. S5D. **E–G**. [^3^H]Deoxyglucose uptake in HL-1 cells (n = 8), aRCMs (n = 6) and hiPSC-CMs (n = 3). **H.** [^14^C]Palmitate uptake in hiPSC-CMs (n = 3). Bar values are means ± SEM. ∗*p* < 0.05.

To investigate whether the beneficial actions of AAs not only apply to lipid oversupply prevention but also to restoration, cardiomyocytes were first exposed for 23 h to HP media, and only the last 1 h to 4∗KLR. This treatment did not lead to v-ATPase reassembly during HP exposure (Figure 5A). Yet, a longer treatment period with 4∗KLR (≥3 h) successfully restored insulin-stimulated glucose uptake (Supplementary Fig. S7). Moreover, when Lys, Leu, and Arg were added together at >20-fold higher concentrations (7 mM, 12 mM, and 10 mM, respectively [[27], [28], [29]]), and then supplemented only during the last 1 h of the total 24 h HP exposure, v-ATPase assembly and activation were successfully restored (Figure 5B–C). This H (igh)-KLR cocktail also restored HP-induced loss of insulin-stimulated AKT and S6 phosphorylation and the loss of insulin-stimulated glucose and fatty acid uptake in cardiomyocytes (Figure 5D–H). Hence, the H-KLR cocktail efficiently normalizes lipid-induced maladaptive abnormalities in v-ATPase dynamics and substrate uptake in a relatively short time.

### 3.7 Effects of AA treatment on v-ATPase dynamics and cardiac parameters in rats fed with a high-fat diet

**Figure 6:**
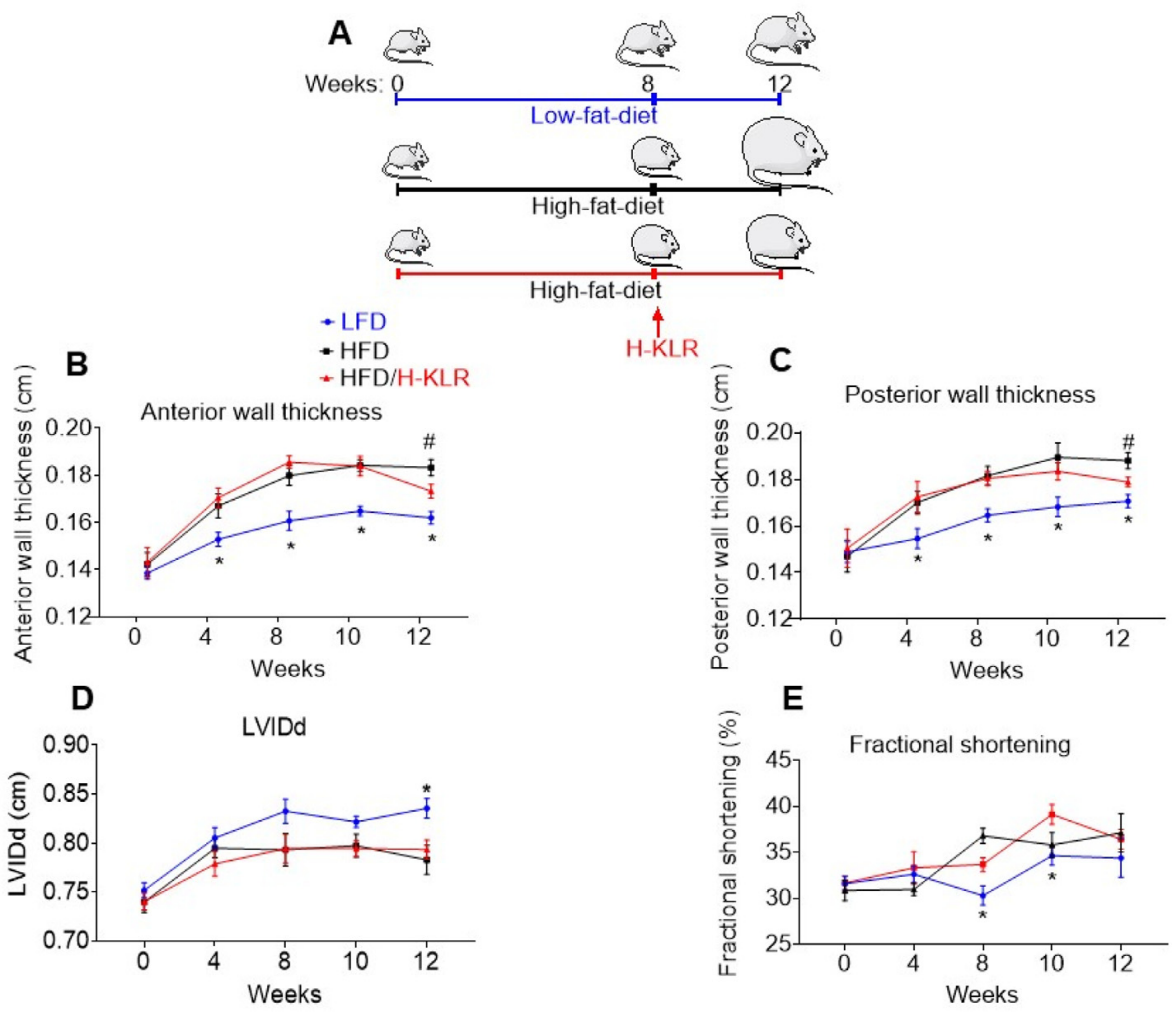
**Combined supplementation of Lys, Leu, and Arg (H-KLR) reverse maladaptive cardiac remodeling in hearts from rats on a cardiomyopathy-inducing high-fat diet (HFD)**. Rats were fed for 12 weeks on a low-fat diet (LFD; 10 en% fat), a high-fat diet (HFD; 60 en% fat), or HFD in combination with high concentrations of Lys (7 mM), Leu (12 mM), and Arg (10 mM) added to the drinking water for the last 4 weeks (HFD/H-KLR). At the end of the diet regime, rats were subjected to echocardiographic measurements. **A.** animal experiment design; **B.** anterior wall thickness in end-diastole; **C.** posterior wall thickness in end-diastole; **D.** LVIDd = left ventricular internal diameter in end-diastole; **E.** fractional shortening. Other echocardiographic measurements are displayed in Supplementary Fig. S7. Graph values are means ± SEM (n = 7). ∗*p* < 0.05 LFD rats vs. HFD rats, #*p* < 0.05 HFD rats vs. HFD/H-KLR rats.

For examining whether H-KLR also normalizes lipid-induced maladaptive cardiac abnormalities *in vivo*, rats were subjected for 12 weeks to a high-fat diet (HFD), and during the last 4 weeks, they were treated with a mixture of Lys/Leu/Arg (7/12/10 mM) in drinking water. After 8 weeks of HFD and at the start of the H-KLR treatment, the rats developed several cardiac abnormalities as established by echocardiography (Figure 6; Supplementary Fig. S8). These abnormalities included increases in anterior and posterior wall thickness of the left ventricle and decreased left ventricular inner diameter, indicating concentric hypertrophy. Furthermore, cardiac function and cardiac output were not (yet) altered (Figure 6; Supplementary Fig. S8). Association between lipid oversupply and this type of cardiac morphological abnormality has been earlier observed in rodents on a lipid-enriched diet [30] and in obese prediabetic participants [31].

**Figure 7:**
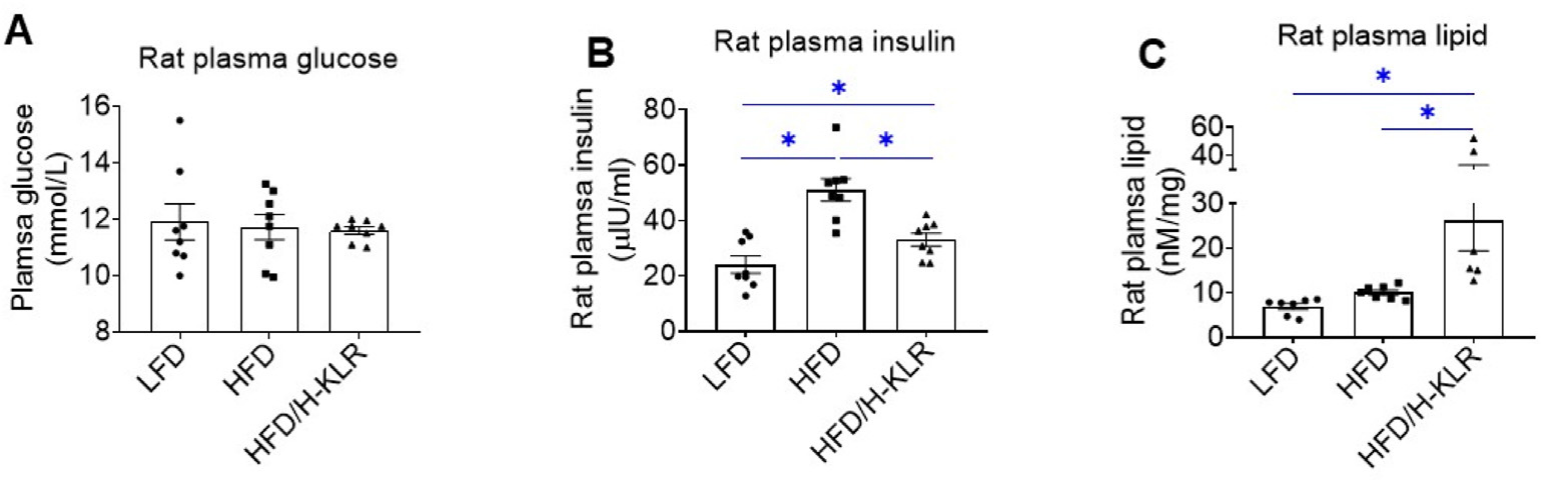
**Combined supplementation of Lys, Leu, and Arg (H-KLR) restore circulating insulin levels in rats fed a high-fat diet**. Rats were fed for 12 weeks a low-fat diet (LFD; 10 en% fat), a high-fat diet (HFD; 60 en% fat), or HFD with high concentrations of Lys (7 mM), Leu (12 mM), and Arg (10 mM) added to the drinking water for the last 4 weeks (HFD/H-KLR). At the end of the 12 weeks' diet regime, blood plasma was taken from the rats for the measurement of **A.** glucose **B**. insulin **C**. triacylglycerol levels. Bar values are means ± SEM (n = 8). ∗*p* < 0.05.

At the end of the diet regime, i.e., after 12 weeks, the HFD group displayed increased body mass and plasma insulin levels, whereas plasma glucose was not altered (Figure 7; Supplementary Fig. S9; Supplementary Table S4), indicative of a prediabetic state. Plasma lipid levels also tended to increase by 1.5-fold. Expression of atrial natriuretic peptide and fibrosis markers, all indicative of advanced diabetes [32,33], were not altered by the HFD regime (Supplementary Table S5). Additionally, plasma levels of all AAs were increased (except for Phe and Trp; Supplementary Table S6). The H-KLR treatment did not alter the HFD-induced increase in body mass but was able to rescue the HFD-induced increase in plasma insulin (Figure 7A and B; Supplementary Fig. S9). However, plasma lipid levels were further increased (Figure 7C). Furthermore, H-KLR treatment lowered the plasma levels of half of the proteogenic AAs in HFD rats almost back to normal levels (Supplementary Table S6). Arg, Leu, and Lys were among the AAs in which concentrations were not decreased in plasma, confirming their supplementation through drinking water. Echocardiography showed that at 12-weeks of HFD, the concentric hypertrophy was at least as pronounced as at 8 weeks HFD (Figure 6; Supplementary Fig. S8). Notably, H-KLR treatment normalized the change in posterior wall thickness (Figure 6D) indicating that part of the hypertrophy had been corrected.

**Figure 8:**
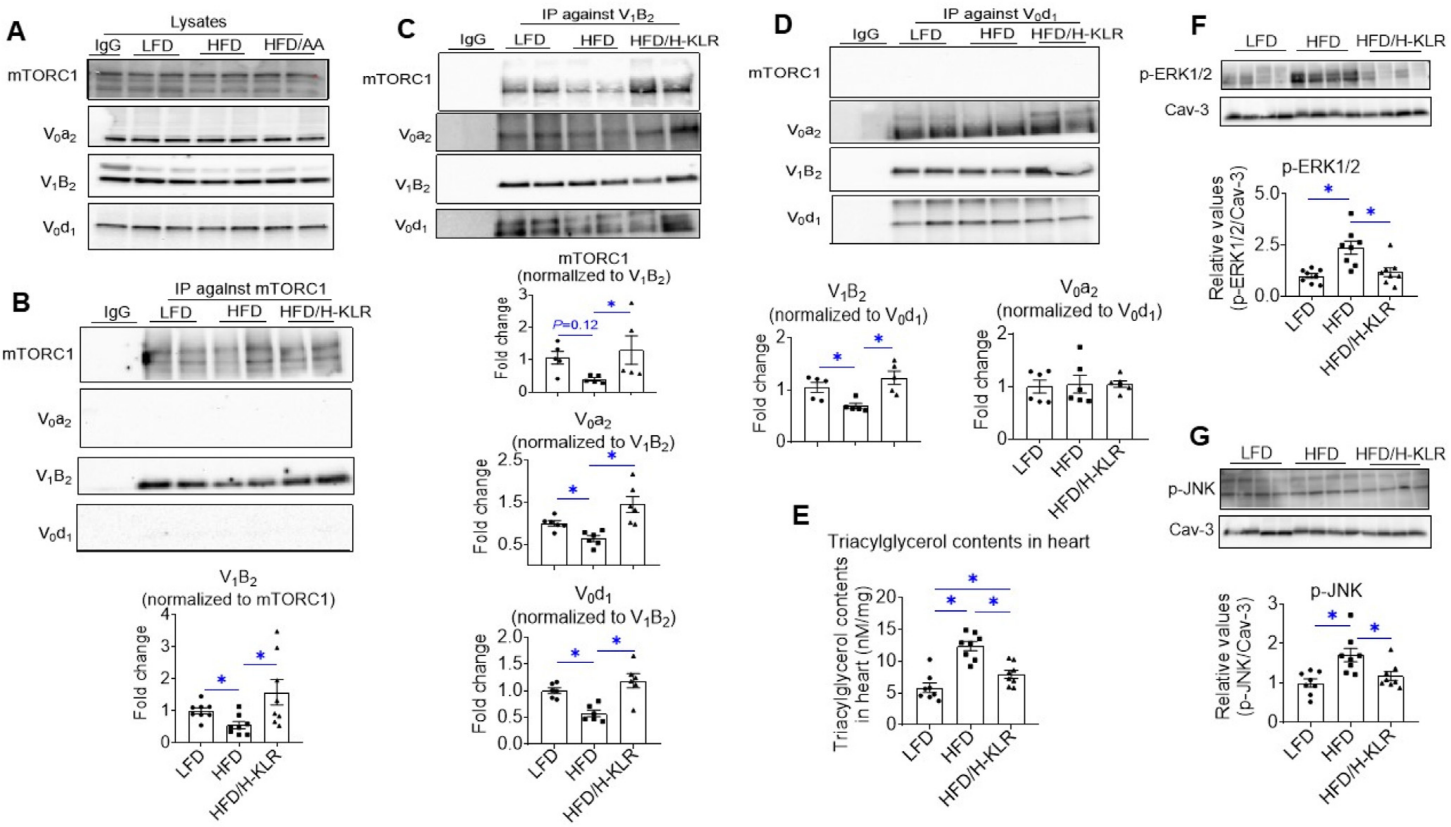
**Combined supplementation of Lys, Leu, and Arg (H-KLR) restores v-ATPase assembly and reverses lipotoxicity in hearts of HFD rats**. Rats were fed for 12 weeks a low-fat diet (LFD; 10 en% fat), a high-fat diet (HFD; 60 en% fat), or HFD with high concentrations of Lys (7 mM), Leu (12 mM), and Arg (10 mM) added to the drinking water for the last 4 weeks (HFD/H-KLR). At the end of the diet regime, hearts were excised and used for lysis and subsequent immunoprecipitation (IP). **A.** Representative western blots of mTOR, V_0_a2, V_1_B2, and V_0_d1 in heart lysates before IP. **B–D.** Co-Immunoprecipitation (Co-IP) of mTOR, V_0_ and V_1_ in heart lysates. **B.** IP against V1–B2 (n = 5). **C.** IP against V_0_-d1 (n = 6). **D.** IP against mTOR (n = 8). Immunoprecipitates were blotted with antibodies against mTOR, V_0_-a2, V_1_–B2, and V_0_-d1, after which the signals were quantified. Representative western blots are displayed. **E.** Assessment of triacylglycerol contents in heart lysates (n = 8). **F–G.** Assessment of lipid-induced signaling actions: representative Western blots and quantifications of basal phosphorylation of ERK1/EKR2 (p-ERK1/2) and JNK (p-JNK) (n = 8). Bar values are means ± SEM ∗*p* < 0.05.

Upon excision and lysis of hearts, v-ATPase binding to mTORC1 and v-ATPase assembly status was determined by immunoprecipitation. Cardiac protein expression of v-ATPase subunits and mTORC1 was not altered by HFD or H-KLR (Figure 8A). In agreement with the *in vitro* data in HL-1 cells (Figure 2), mTORC1 associated with the v-ATPase V_1_-subcomplex in the LFD condition, but did not bind to the V_0_-subcomplex in any condition (Figure 8B–D). The HFD regime decreased mTORC1 binding to the V_1_-subcomplex and also induced the disassembly of V_0_/V_1_ (Figure 8B–D), in agreement with the *in vitro* data using HP-exposed cardiomyocytes (Figure 2). This was entirely reversed by the H-KLR treatment (Figure 8B–D).

The heart lysates were also used for the assessment of myocellular triacylglycerol content and lipid-induced signaling actions, such as phosphorylation of ERK1/2 and JNK. These parameters were all increased upon HFD, but markedly reversed by the H-KLR treatment (Figure 8E–G). Taken together, the *in vivo* H-KLR treatment effectively restored v-ATPase dynamics and lipid metabolism in the lipid-overloaded heart.

## 4 Discussion

For a long time, v-ATPase was merely regarded as a proton pump mediating the luminal acidification of subcellular organelles, but in the last decade, it became evident that this protein complex is also an essential component in mTORC1 activation by AAs [34]. However, in this AA sensing pathway, the established mechanism of v-ATPase regulation, being v-ATPase cycling between assembled and/disassembled states, has been disregarded. Moreover, the possibility of reciprocal activation of v-ATPase by mTORC1 has not yet been considered.

In the present study, we made three main observations: (4.1) The AA mix of Lys (K), Leu (L), and Arg (R) reactivates v-ATPase-mediated proton pumping during lipid overload, and the underlying mechanism includes endosome/lysosome-centric AA sensing by mTORC1. (4.2) The KLR cocktail prevents and reverses lipid accumulation, insulin resistance, and contractile dysfunction *in vitro* in lipid-overloaded cardiomyocytes. (4.3) Supplementation of these three AAs *in vivo* to rats on HFD induces v-ATPase reassembly, reduces maladaptive lipid accumulation, and partly reverses HFD-induced concentric remodeling. These observations are further discussed below.

### 4.1 Selection of the most potent AAs to activate v-ATPase and their combined mechanism of v-ATPase reactivation during lipid overload

The effect of AAs on v-ATPase activity has been investigated earlier, but an opposite action has been reported, i.e., an inhibition [23]. In that respective study, all AAs were added in a cocktail at their 1x concentration, under which conditions we also observed v-ATPase inhibition in cardiomyocytes (Figure 1A). We report here for the first time that higher AA concentrations (4x higher than low physiological), do have a stimulatory action on v-ATPase. Next, at these 4∗ concentrations we selected the three most potent AAs to stimulate v-ATPase. It may not be a coincidence that among these three AAs (Leu/Lys/Arg), there are two basic AAs, which will accumulate into endosomes/lysosomes because of weak base trapping. Subsequently, Arg and Lys would activate mTORC1 through the endosome/lysosome-centric inside-out-mechanism of AA sensing, depending on SLC38A9 [13]. With respect to Leu, this AA has been generally regarded as one of the most potent mTORC1 activators, whose effect is mediated using the adaptor protein Sestrin2, with a high affinity for Leu [35]. Upon Leu binding, Sestrin2 dissociates from the GATOR2 complex, a positive regulator of mTORC1 [36]. Hence, endosomal and cytoplasmic sensors may cooperate in response to the 4∗KLR cocktail to effectively activate mTORC1. Subsequently, mTORC1 binds to the cytoplasmic v-ATPase V_1_ subcomplex. The immunoprecipitation experiments shown in Figure 2 indicated that this is a crucial step in the reassembly of v-ATPase. Arguably, a structural change within V_1_ may be required to enable the subcomplexes to reassemble. Such a structural change could perhaps be induced by unknown phosphorylation of one of the V_1_ subunits by mTORC1. This step is also dependent on Ragulator, an essential component in the complex formation between mTORC1 and v-ATPase [36].

For completion, we wish to mention that AA combinations other than KLR could also have been effective in synergistically activating mTORC1: for instance, a combination of glutamate with isoleucine, given that each of these AAs stimulated v-ATPase activity to a comparable magnitude as Lys, Leu, or Arg. However, Glu and Ile were not selected because these stimulatory effects were not statistically significant (P > 0.05). Interestingly, Glu has been reported to stimulate mTORC1 in intestinal cells [37] and Ile could act in this respect using the same mechanism as Leu.

Ultimately, this AA-induced mTORC1-mediated mechanism of v-ATPase activation will lead to the acidification of endosomes. This AA-induced acidification is especially beneficial under conditions of lipid overload to overcome the maladaptive deacidification of endosomes induced by lipids.

### 4.2 AA treatment prevents and restores insulin resistance and contractile dysfunction in lipid-overloaded cardiomyocytes

The AA-induced reacidification of endosomes is at the start of a beneficial series of events. First, the reacidified endosomes can again serve as storage compartments for CD36, enabling a net reinternalization of CD36 from the cell surface; thereby reducing myocellular lipid uptake and accumulation. The resulting intracellular lowering of lipid metabolites relieves the block on insulin signaling and insulin-stimulated substrate uptake. Ultimately, the 4∗KLR treatment leads to the preservation of contractile function.

Importantly, the KLR mixture not only prevented the loss of insulin-stimulated glucose uptake in lipid-overloaded cardiomyocytes (when added simultaneously with the onset of the HP culturing), but also fully repaired impaired insulin-stimulated glucose uptake (i.e., when added after the HP culturing had led to the loss of insulin-stimulated glucose uptake). Repair of insulin-stimulated glucose uptake by the KLR mixture is already observed at relatively modest concentrations of Lys, Leu, and Arg (i.e., 4x), but increasing their respective concentrations by > 20-fold markedly shortened the repair time.

### 4.3 AA treatment normalizes v-ATPase and lipid dynamics in hearts from high-fat diet-fed rats, and restores cardiac pathophysiological adaptations

The *in vitro* therapeutic actions of the KLR cocktail prompted us to additionally study the *in vivo* therapeutic potential, using rats fed with HFD. We supplemented the HFD for 8 weeks before starting the 4-weeks H-KLR treatment, based on our earlier findings that hearts from rats on 8 weeks HFD displayed loss of endosomal acidification, CD36 translocation to the cell surface, and insulin resistance [9]. After 8 weeks of HFD, we observed alterations in cardiac morphology indicative of the development of concentric hypertrophy, which was also prominently present after 12 weeks. Cardiac hypertrophy often precedes the development of heart failure [38], and it is known that a prolonged HFD regime (>20 weeks) additionally results in decreased cardiac function [39,40]. Hence, the rats at 12 weeks of HFD are on a one-way road towards lipid-induced cardiomyopathy. Importantly, and in accordance with the *in vitro* beneficial effects, the KLR supplementation normalized the mTORC1–v-ATPase binding and the associated v-ATPase assembly status in the hearts of HFD rats. As a result, maladaptive lipid accumulation and lipid-induced signaling were normalized; and also the maladaptations in anterior and posterior wall thickness. In the *in vitro* studies with lipid-overloaded cardiomyocytes, we causally linked the beneficial effects of the KLR treatment on insulin sensitivity and contractile function to the reactivation of the mTORC1–v-ATPase axis (i.e., with pharmacological inhibition by rapamycin and BafA, along with silencing of V_1_–B2 and Lamtor1); and hence, it can be inferred that this reactivation mechanism is also a critical factor in the beneficial effects of the KLR mixture *in vivo* on cardiac lipid profile and morphology in HFD rats.

## 5 Conclusions

In the present study, we have unmasked the AA Lys, Leu, and Arg as potent regulators of lipid metabolism by the inhibition of subcellular CD36 translocation. We also identified the underlying molecular mechanism of SLC38A9/Lamtor1-mediated mTORC1 activation followed by v-ATPase-V_1_ binding and subsequent reassembly with v-ATPase-V_0_, leading to endosomal reacidification and CD36 internalization. Furthermore, we substantiated the disclosed molecular mechanism *in vivo* and showed that the 4∗KLR cocktail rescues cardiac lipid-induced hypertrophy. Based on extensive similarities in the regulation of CD36 translocation between heart and skeletal muscle, the applied 4∗KLR cocktail may have similar effects on mTORC1–v-ATPase activation in skeletal muscle compared to the heart. This would imply that 4∗KLR treatment may not only resolve lipid-induced cardiac maladapatations, but also lipid-induced insulin resistance and diabetes at the whole-body level. In accordance with this, the 4∗KLR treatment restored plasma insulin and partially plasma AA concentrations to their basal levels in HFD rats (Figure 7; Supplementary Table 6). However, the improvement of insulin resistance by the 4∗KLR treatment using CD36 internalization in muscle comes at a cost. However, keeping the lipids out of the muscle cells would be expected to cause an extra rise in circulating lipids in the HFD rats, which is observed. It remains to be examined whether the beneficial KLR actions outweigh this possible negative side effect. Moreover, we cannot exclude that part of the observed beneficial effects of H-KLR that can be attributed to the effects of these AAs on organs other than the heart, which does not take away its potential use for the treatment of lipid overload.

Remarkably, mTORC1 has a negative connotation in type-2 diabetes research, because its activation by insulin mediates a negative feedback loop in insulin signaling, likely contributing to myocellular insulin resistance. Specifically, activated mTORC1 reduces signaling through insulin-receptor-substrate-1 (IRS1) by inhibitory Ser-phosphorylations [41,42]. To resolve this apparent paradox between the poor reputation of mTORC1 in the lipid overloaded insulin-resistant heart and the potential beneficial use of mTORC1 activation as a therapeutic strategy to cure lipid-induced cardiomyopathy, we speculate the following: While under basal conditions, mTORC1 is localized at the endosomal membranes (Figure 2A), and it has to migrate into the cytoplasm to gain access to IRS1 for performing the inhibitory Ser-phosphorylation [43]. Moreover, when activated by AA, and not by insulin, mTORC1 may remain attached to v-ATPase in endosomal membranes so that the inhibitory phosphorylation event of proximal insulin signaling will not occur.

Finally, the present findings as observed in rodents may extend to the human setting, considering that the KLR cocktail also prevents lipid-induced insulin resistance in hiPSC-CMs by v-ATPase1 reassembly – offering the potential of these AAs to be used as nutraceuticals to limit lipid uptake in diabetics with cardiomyopathy.

## Author contributions

JL, DN, MN, and JG designed the experiments; SW, FS, LW, A Sun, FN, A Strzelecka, and UC performed the experiments; SW and JL analyzed the data; MZ was involved in the design of statistical analyses; SW, JL, DN, MN, and JG wrote the article. All authors discussed the data and commented on the study before submission.

## Funding

SW and A Sun received support from the 10.13039/501100004543China Scholarship Council. MN is the recipient of a Dutch Heart Foundation Dekker grant, nr. 2019T041 and a 10.13039/501100001826ZonMw Off Road grant, nr. 04510011910065. This study was also supported by the Netherlands Organisation for Scientific Research (NWO-ALW grant nr. ALWOP.367 to JL).
