# Supplementary material for: Specific amino acid supplementation rescues the heart from lipid overload-induced insulin resistance and contractile dysfunction by targeting the endosomal mTOR–v-ATPase axis
Source: Mol Metab. 2021 Jul 13;53:101293. doi: 10.1016/j.molmet.2021.101293 (PMC8350375; doi:10.1016/j.molmet.2021.101293)

## Slide 1
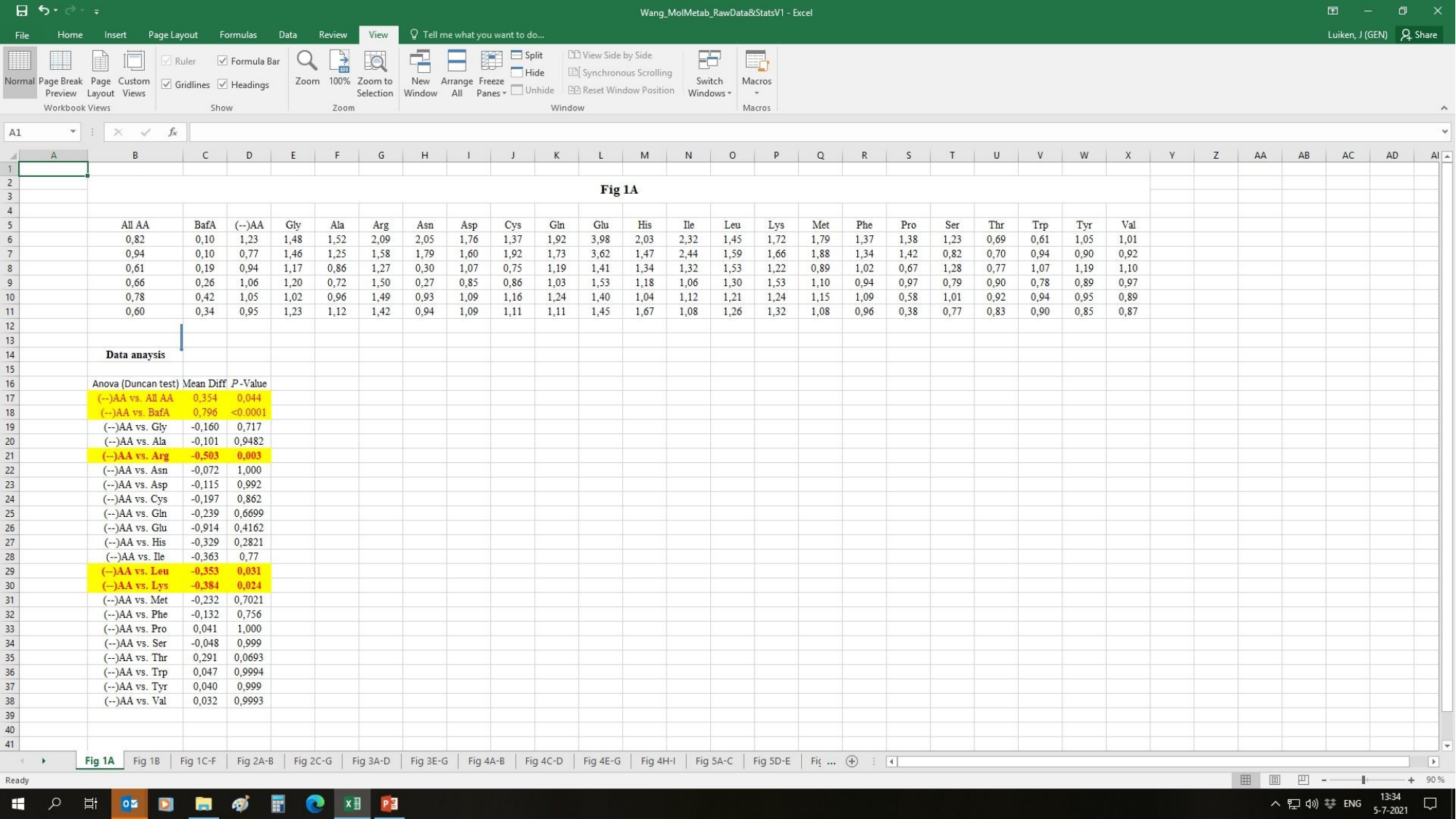

## Slide 2
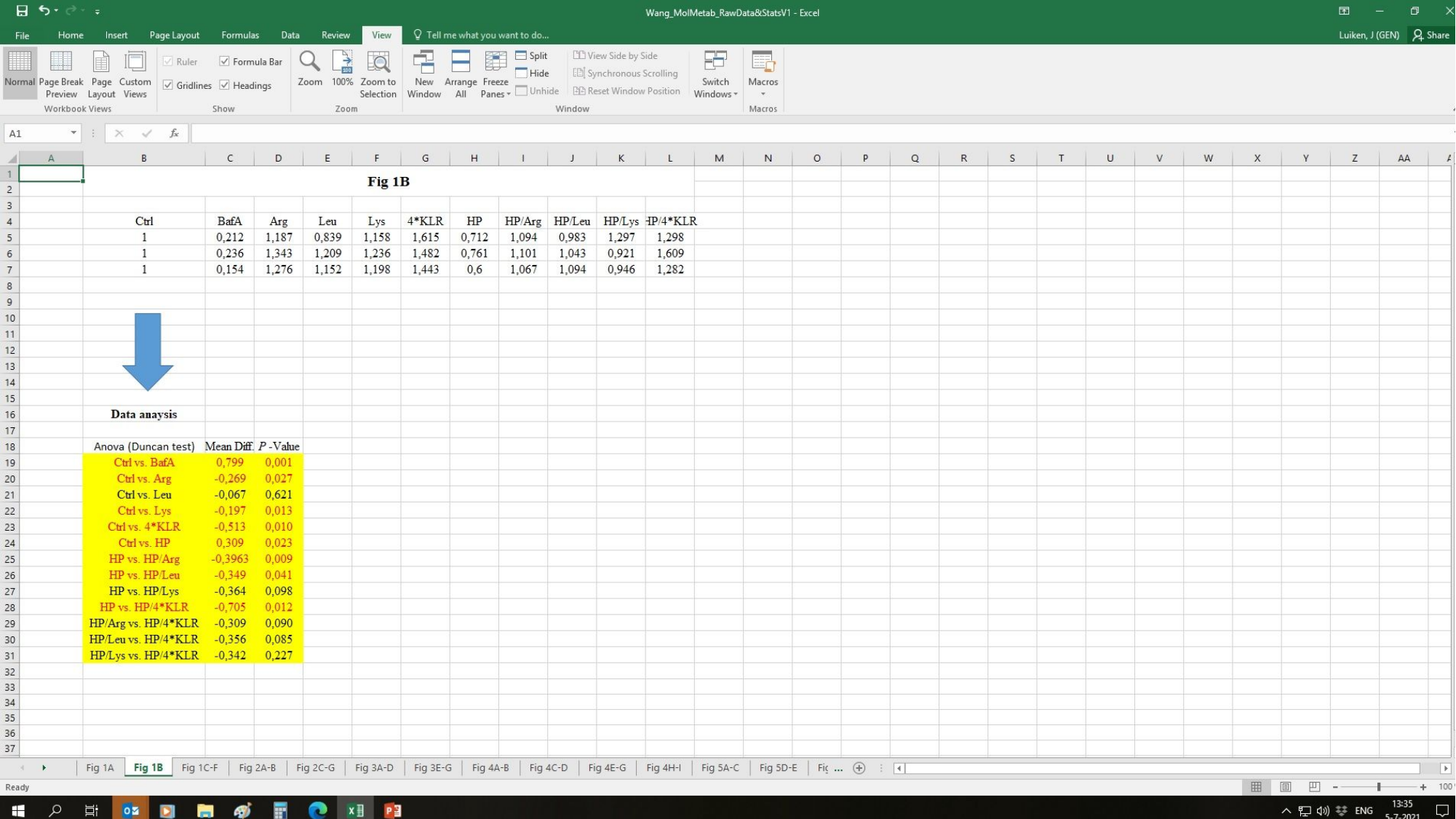

## Slide 3
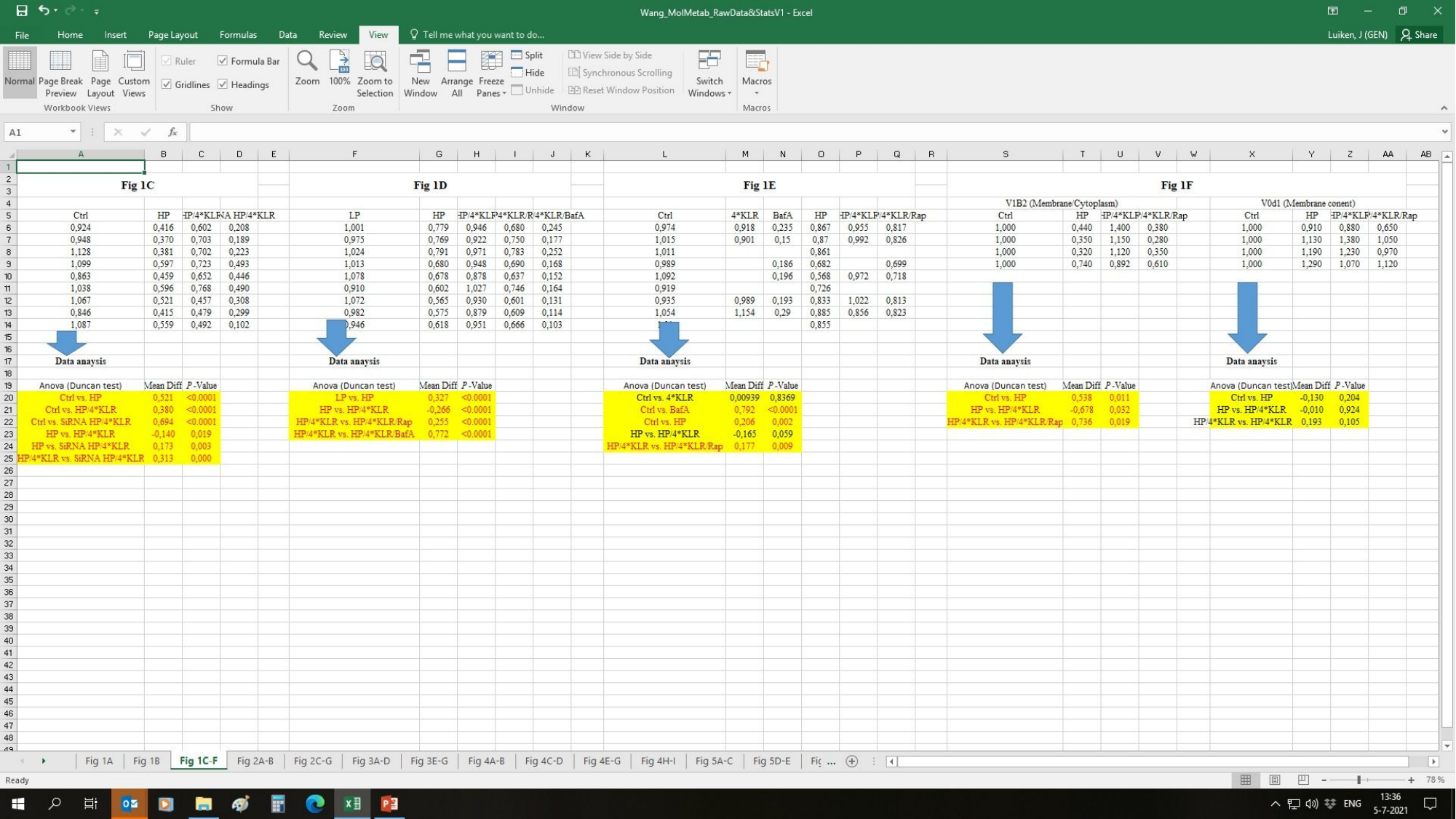

## Slide 4
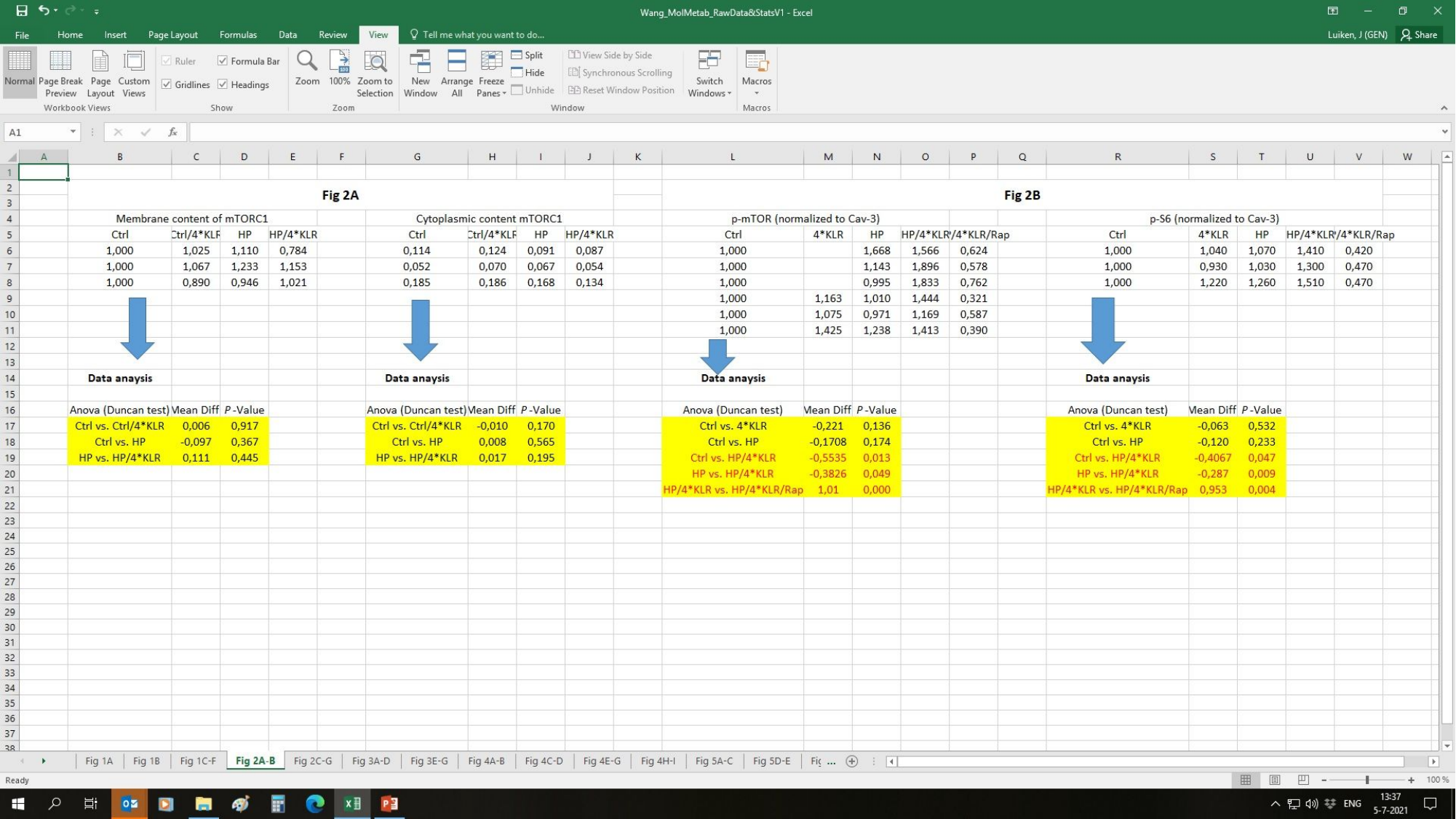

## Slide 5
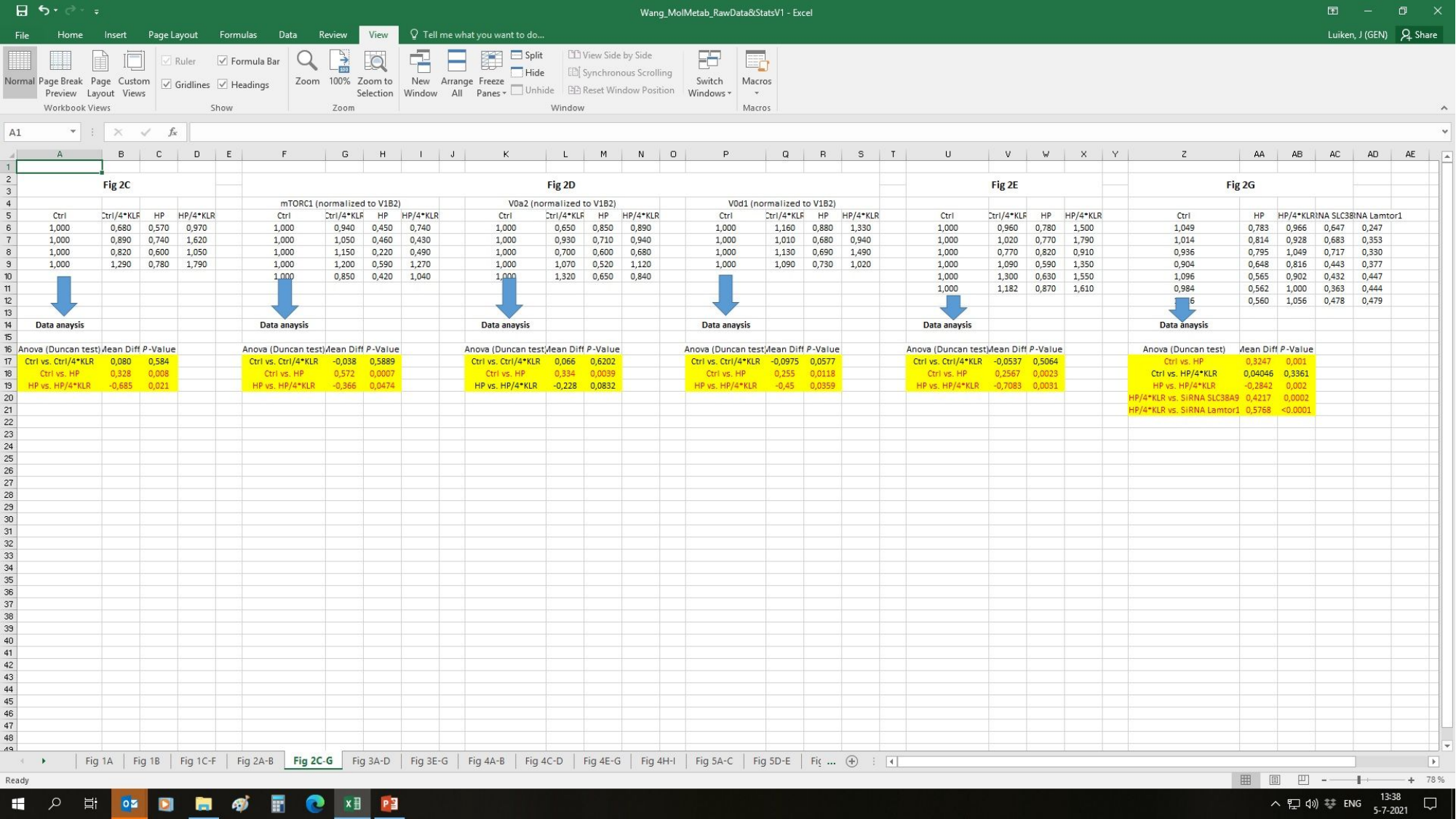

## Slide 6
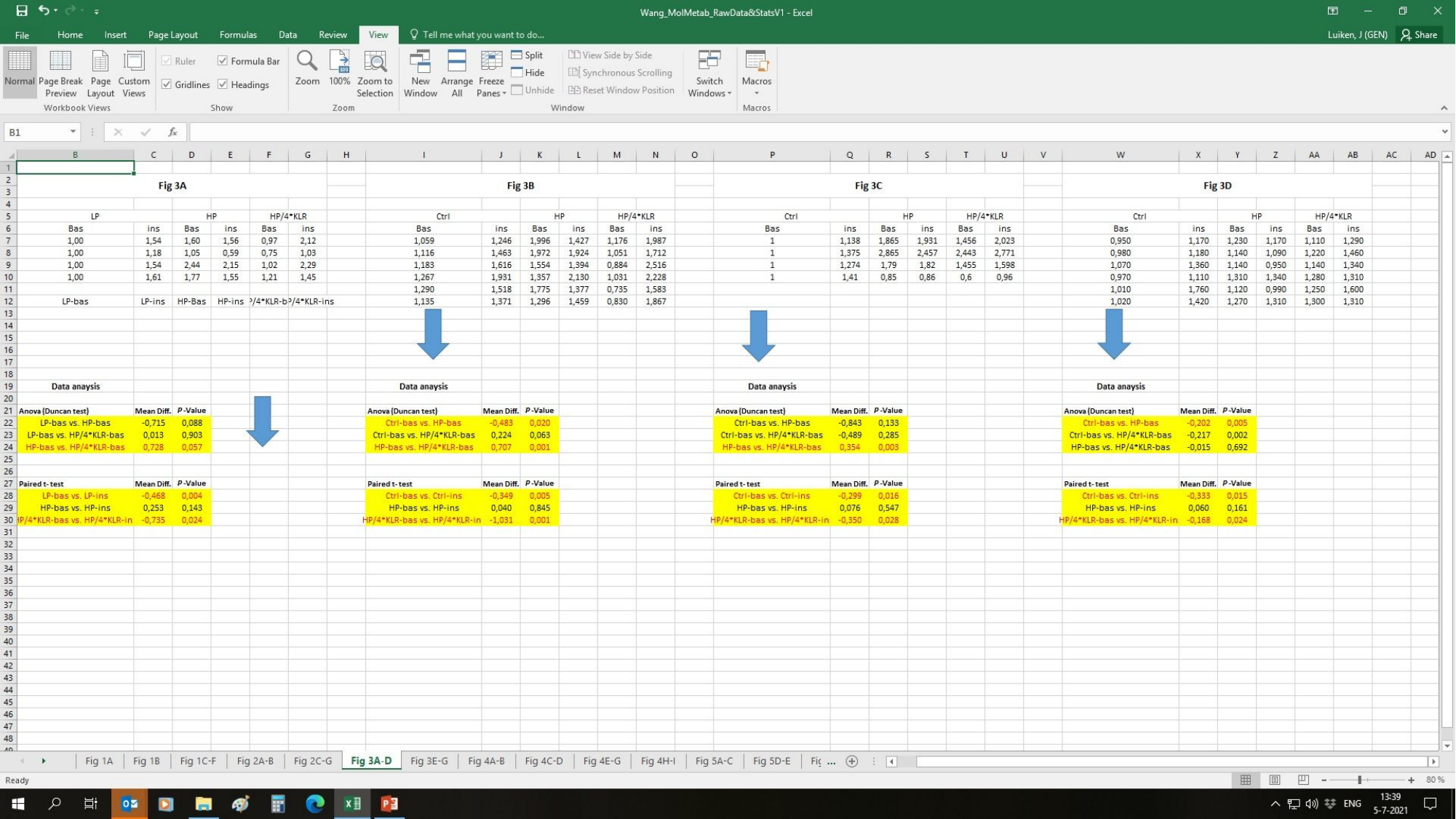

## Slide 7
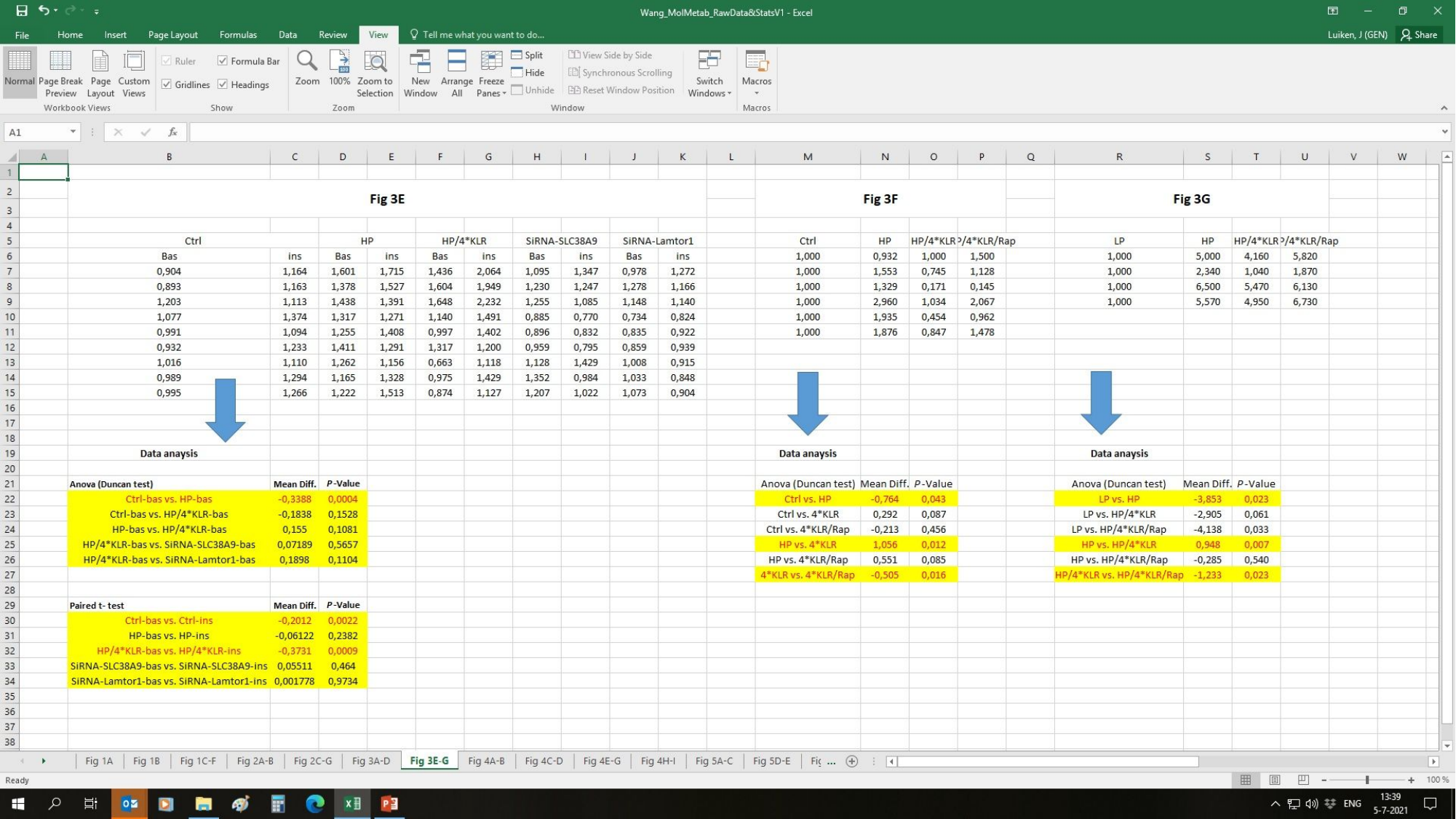

## Slide 8
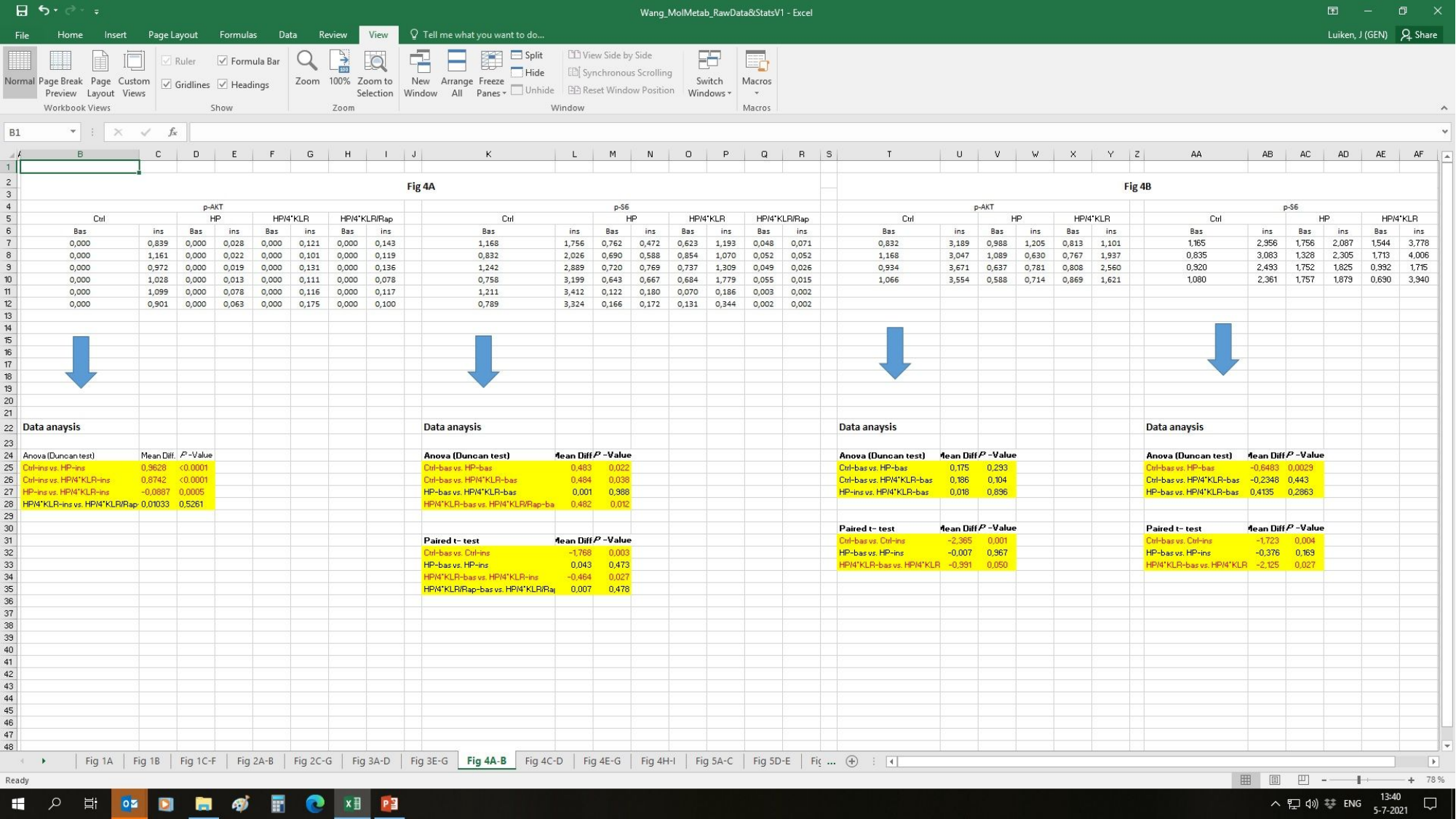

## Slide 9
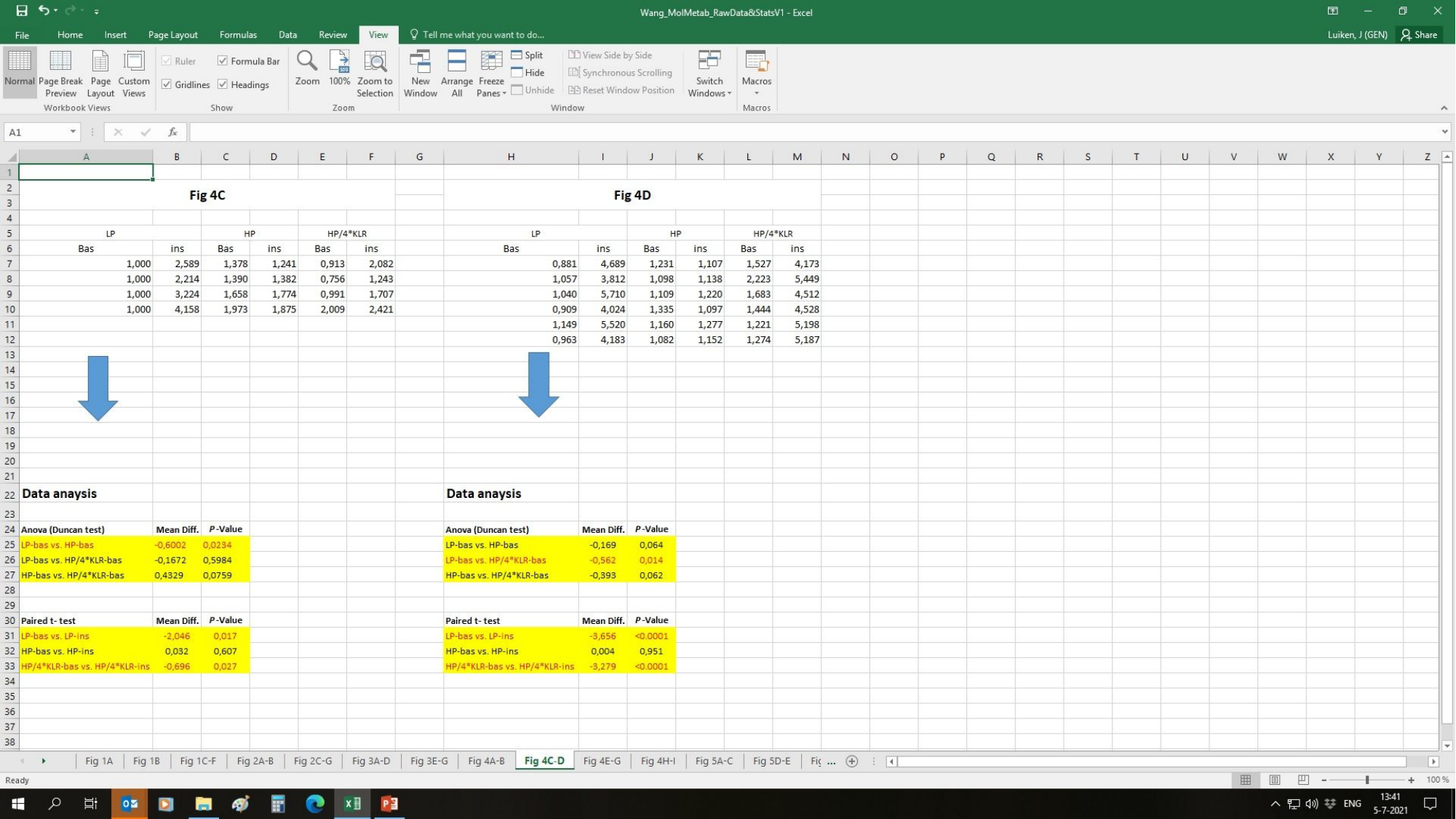

## Slide 10
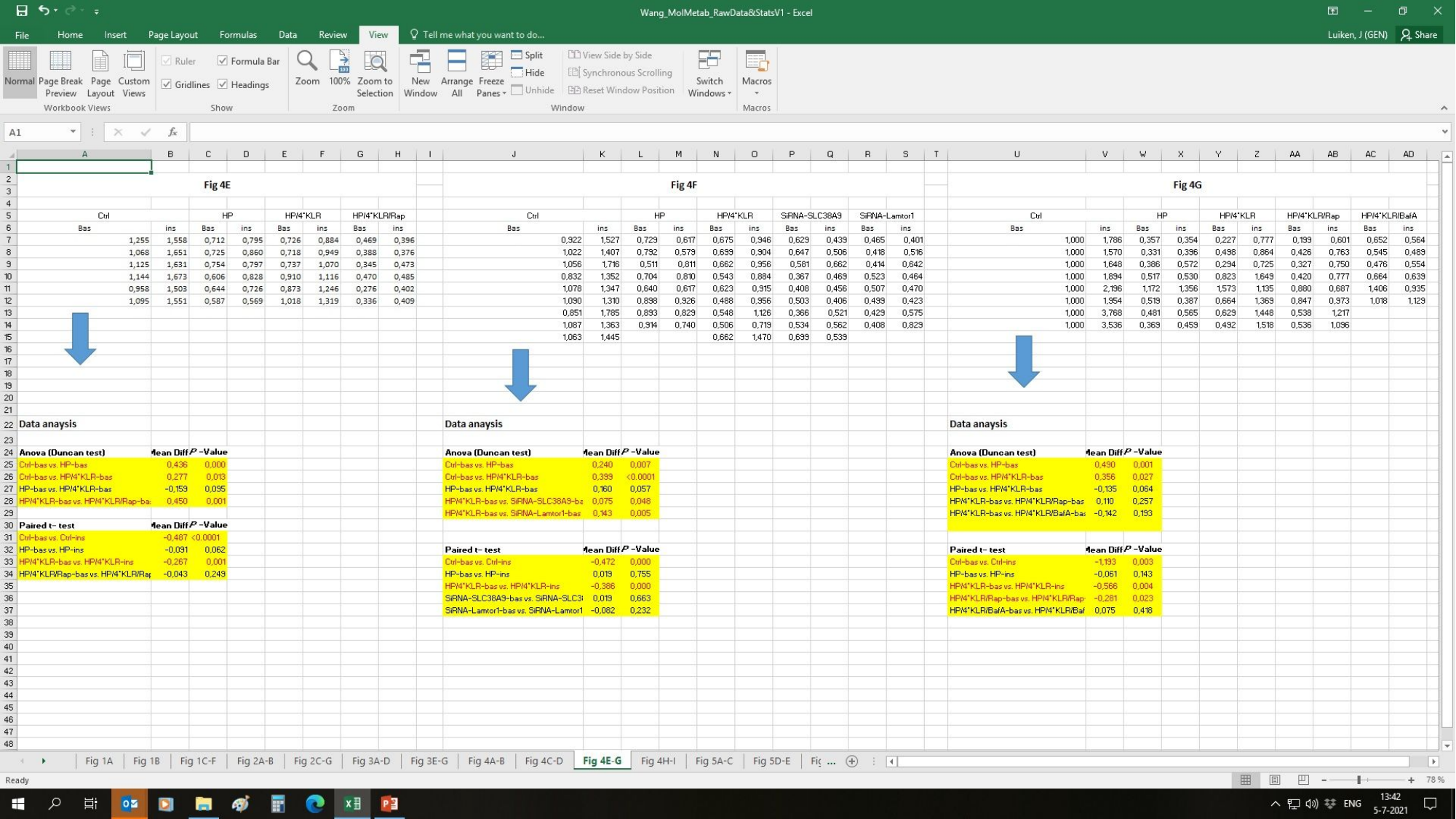

## Slide 11
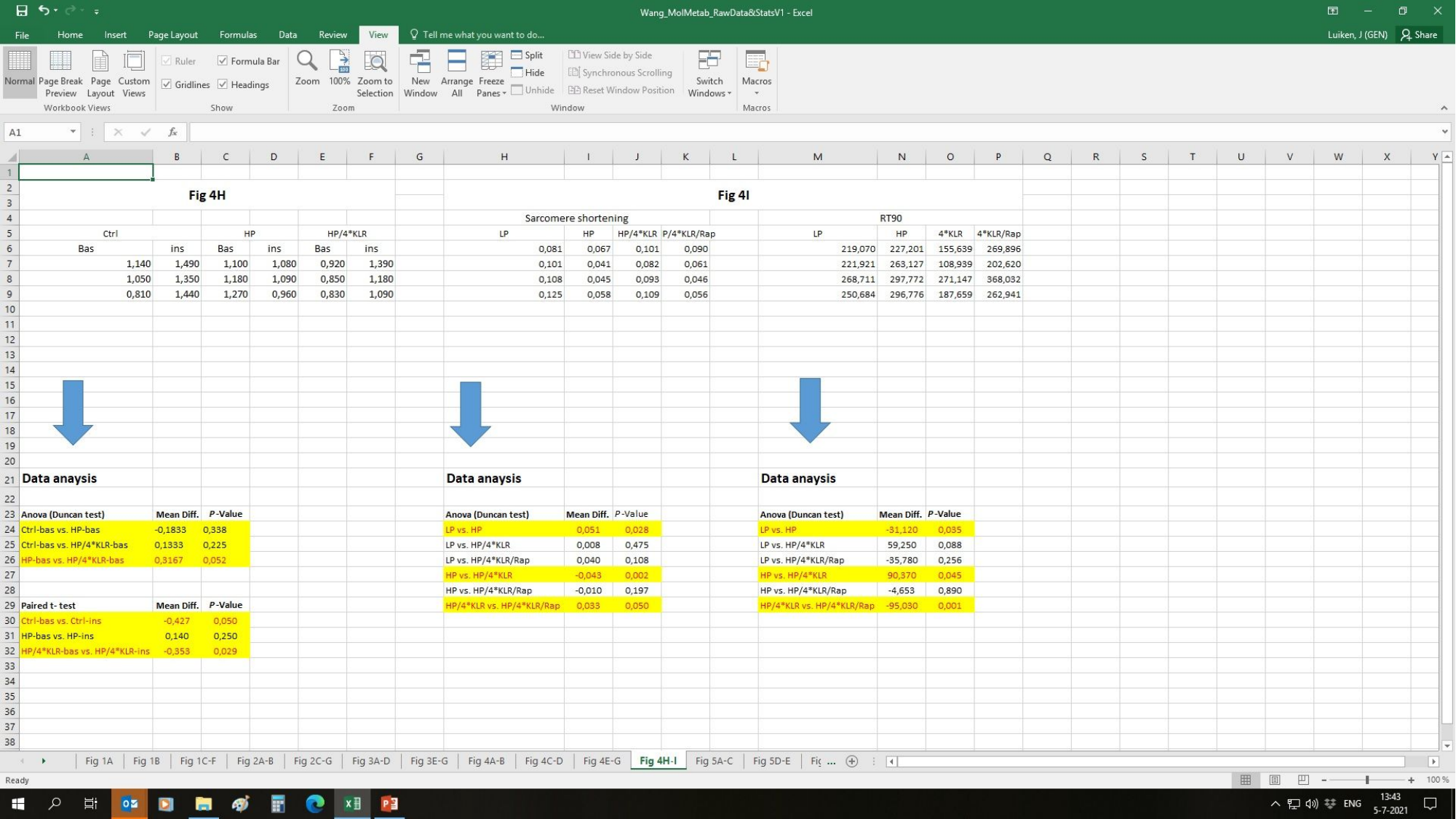

## Slide 12
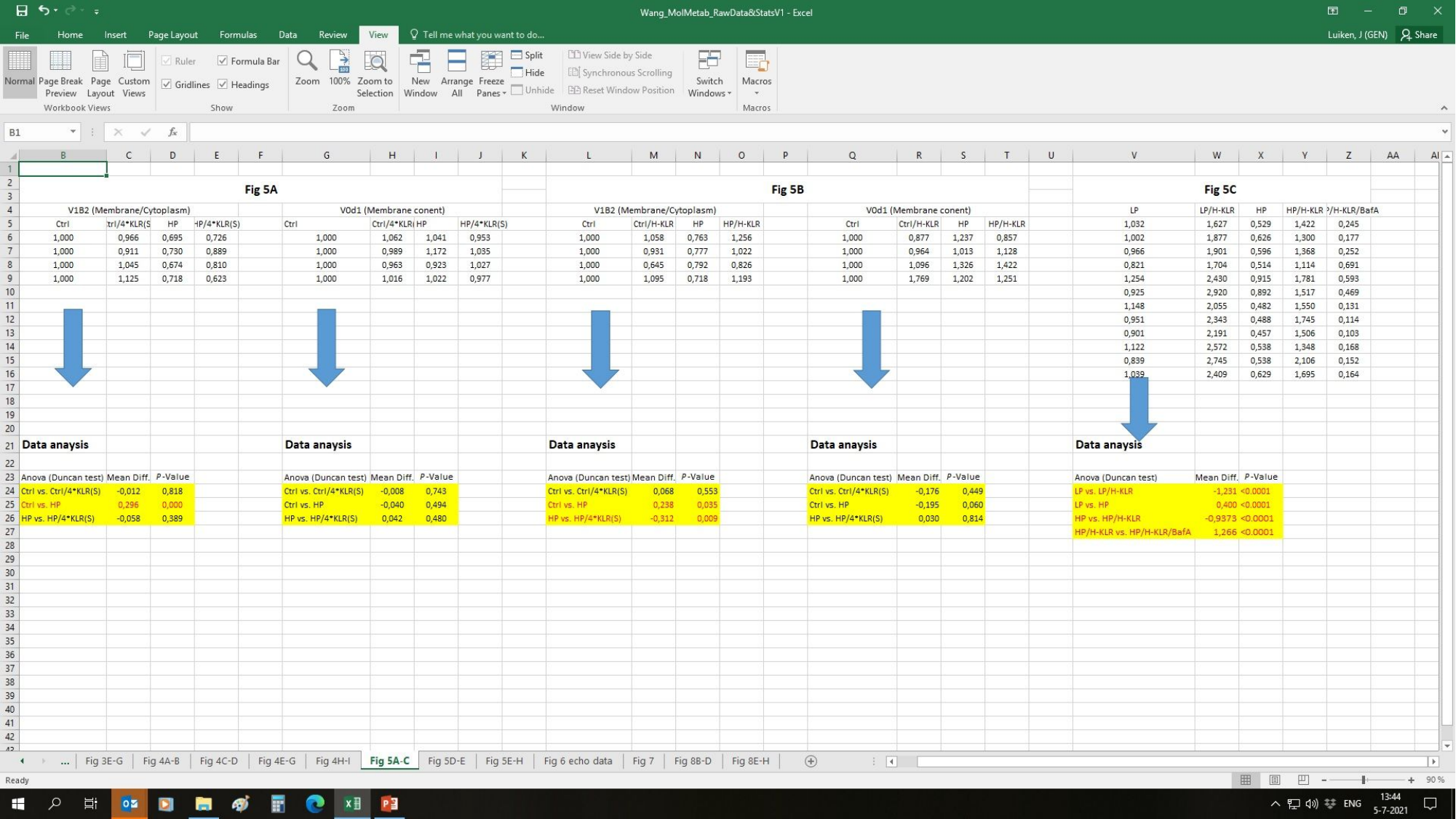

## Slide 13
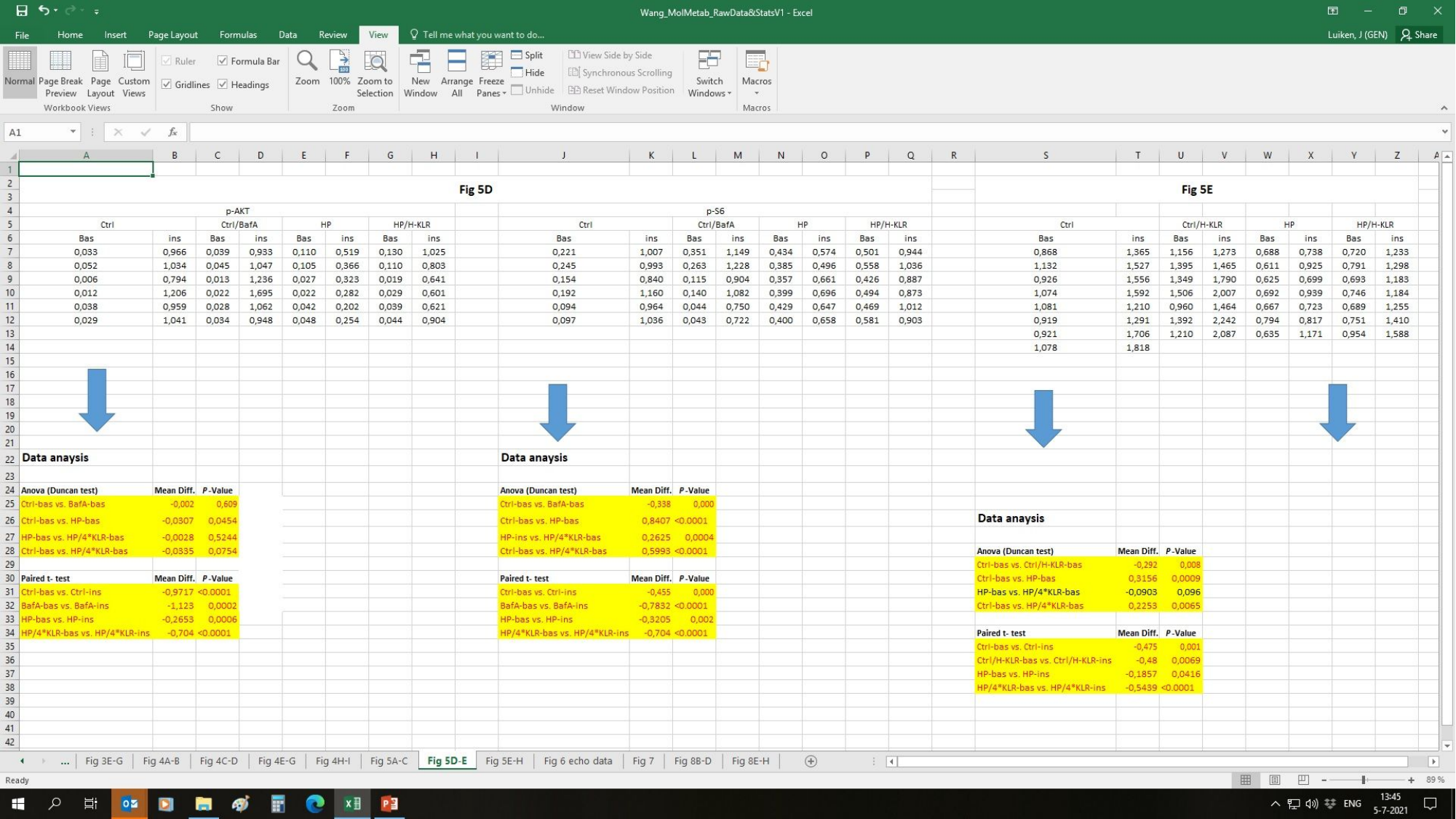

## Slide 14
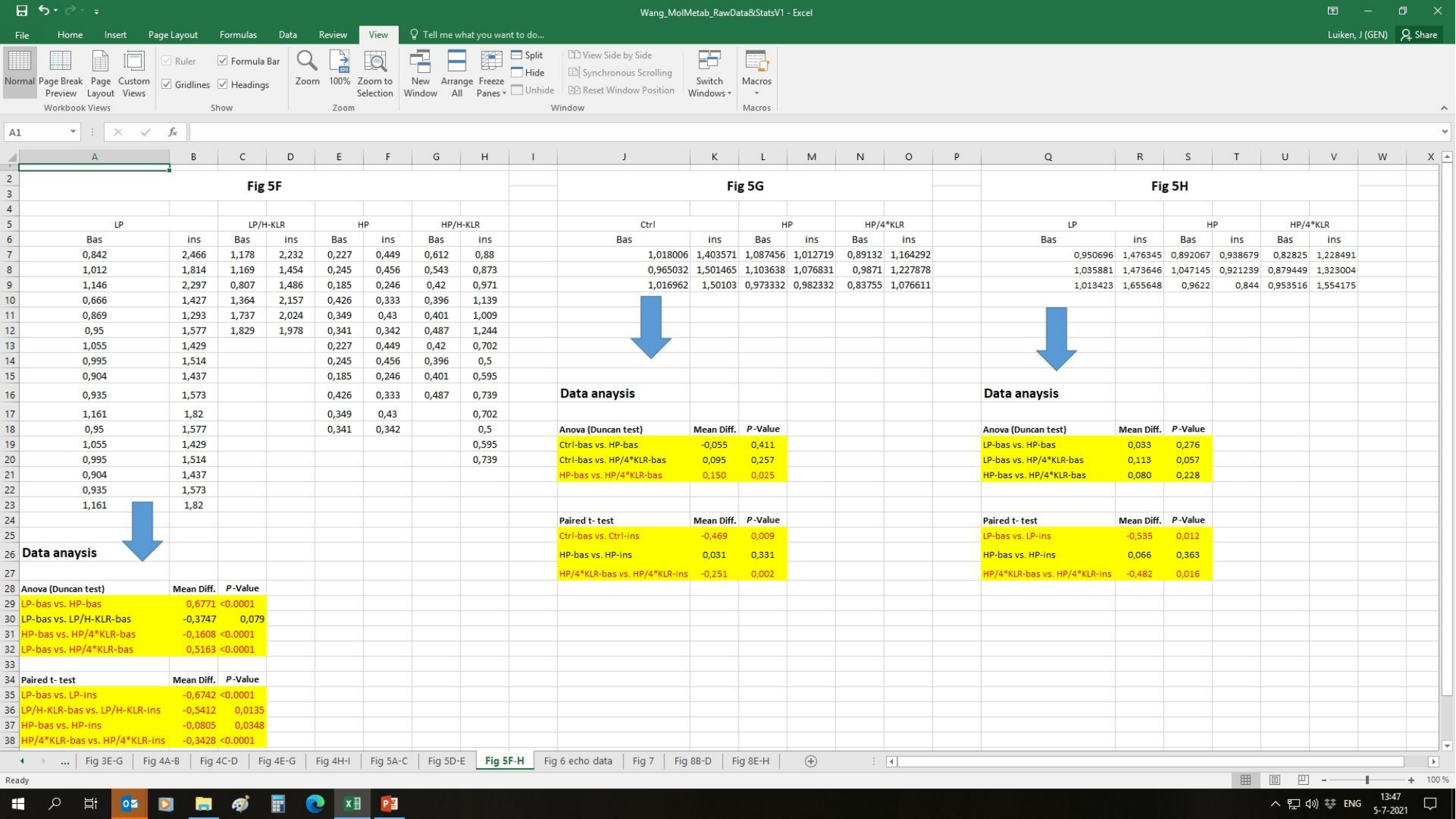

## Slide 15
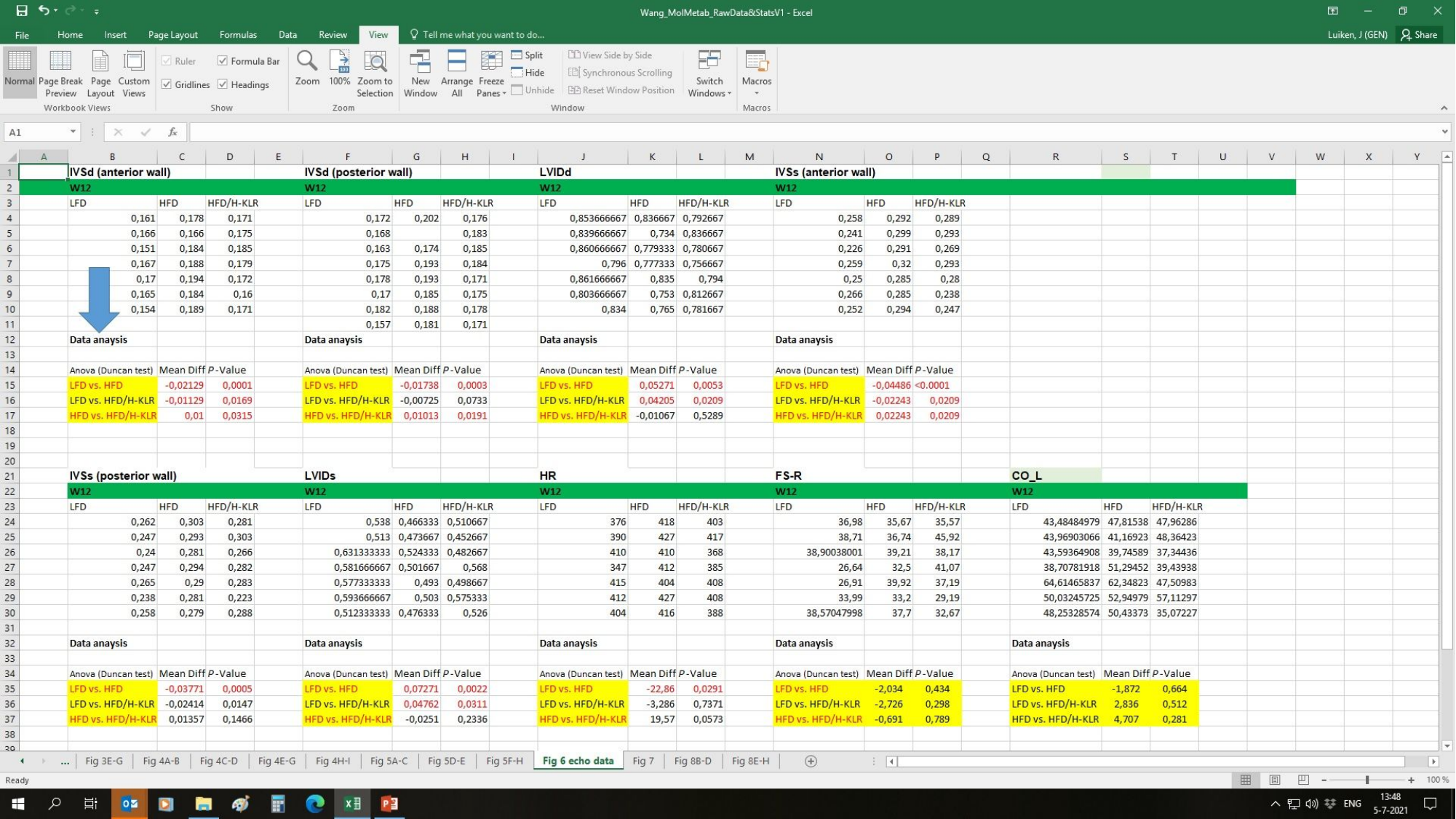

## Slide 16
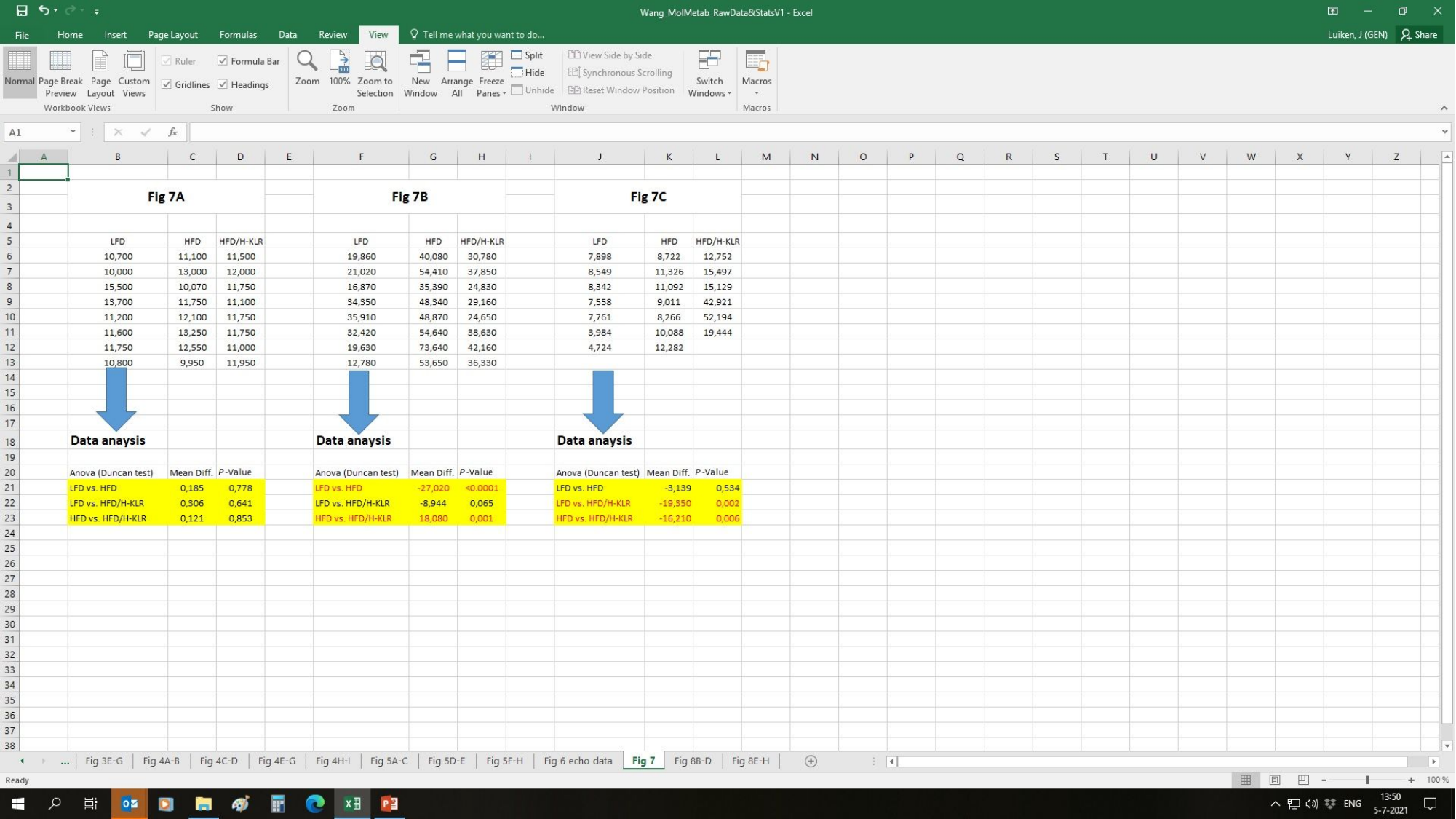

## Slide 17
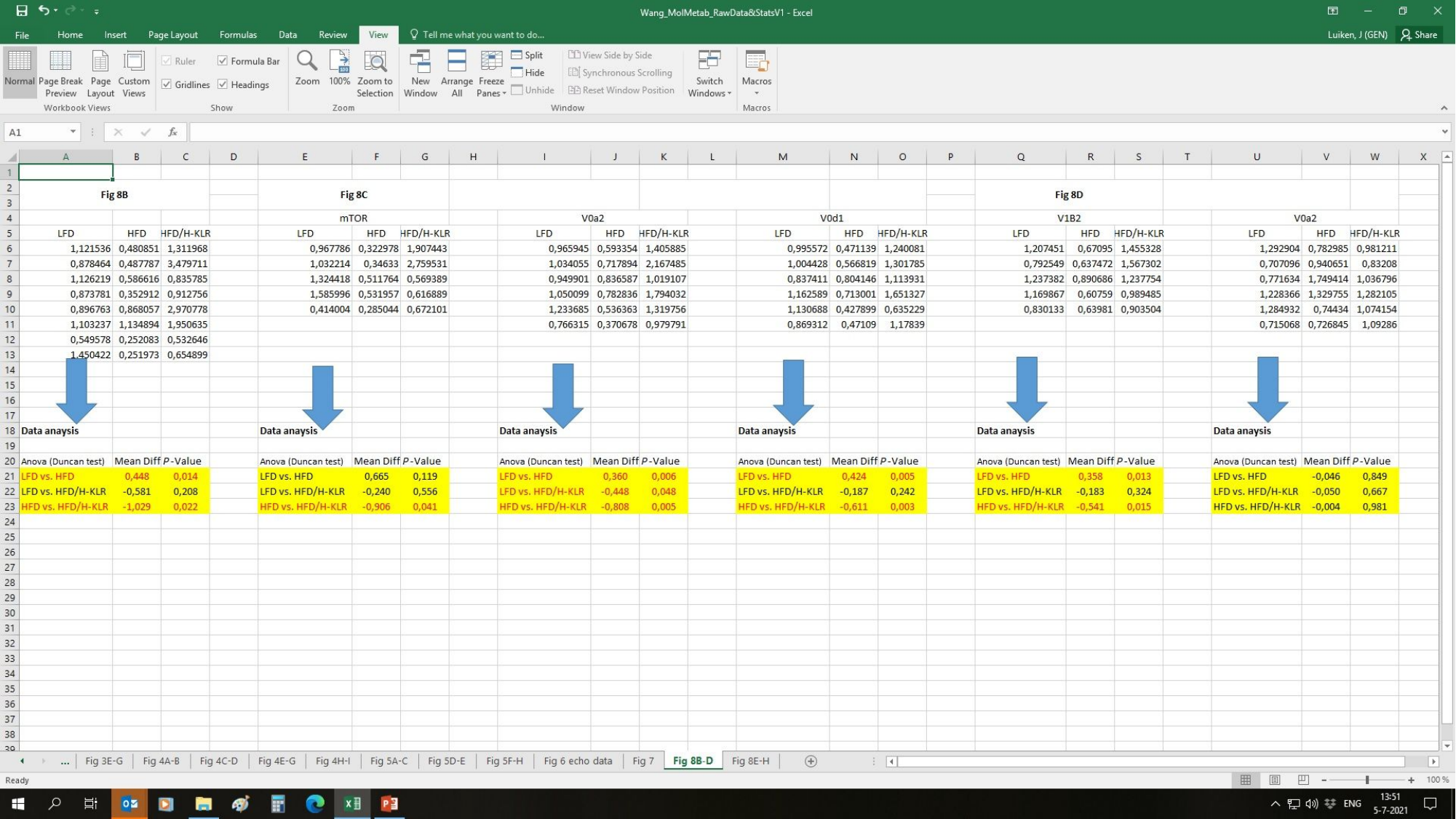

## Slide 18
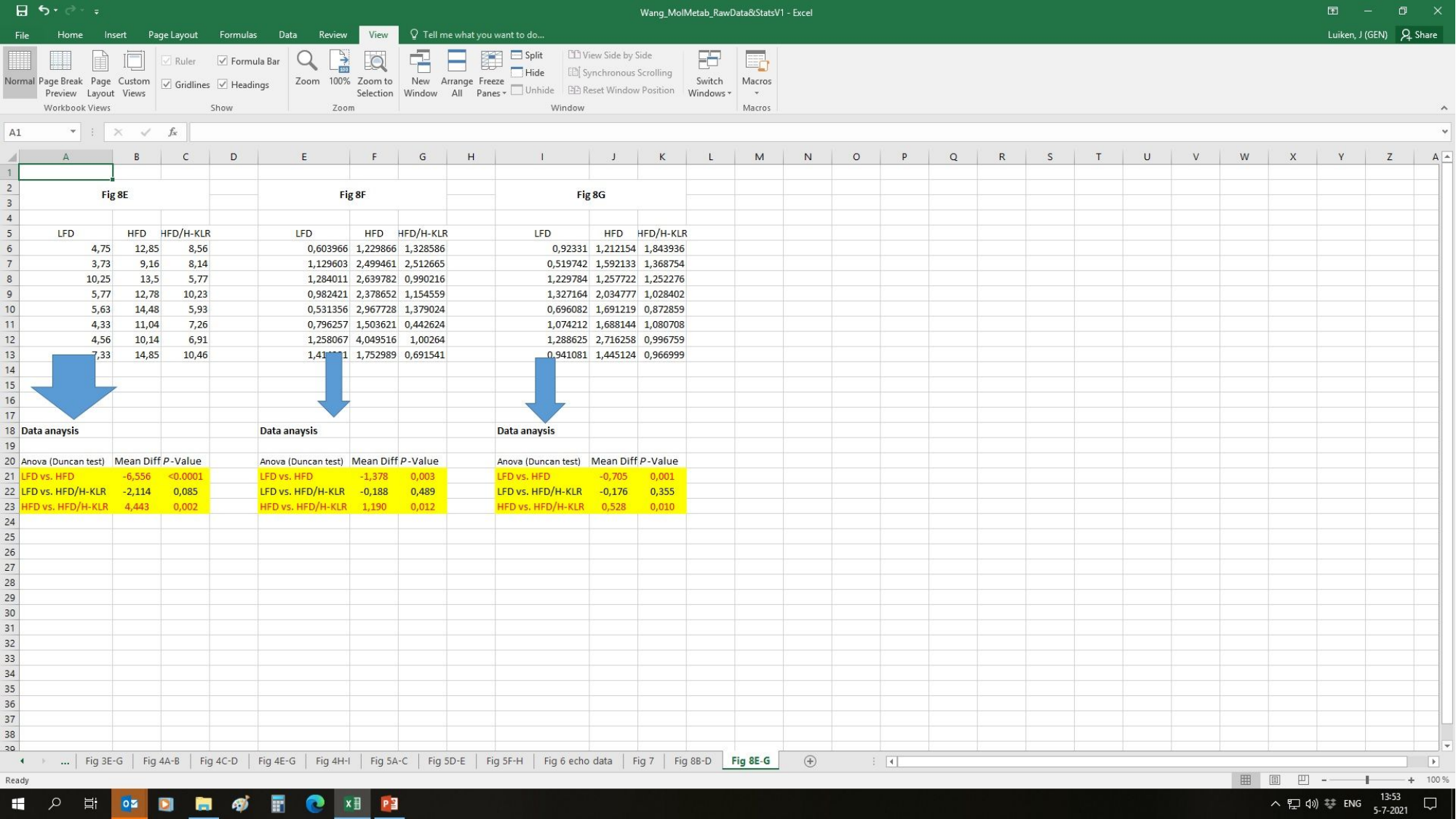

Supplement: Multimedia component 1 [file mmc1.pptx]
