# Supplementary material for: Specific amino acid supplementation rescues the heart from lipid overload-induced insulin resistance and contractile dysfunction by targeting the endosomal mTOR–v-ATPase axis
Source: Mol Metab. 2021 Jul 13;53:101293. doi: 10.1016/j.molmet.2021.101293 (PMC8350375; doi:10.1016/j.molmet.2021.101293)
Supplement: Multimedia component 2 [file mmc2.docx]

**Supplementary Figures, Methods, and Tables**

**Article:**

**Title: Specific amino acid supplementation rescues the heart from lipid overload-induced insulin resistance and contractile dysfunction by targeting the endosomal mTOR–v-ATPase axis**

**Authors: Shujin Wang *et al.***

**Figure S1 (related to Figure 1A): AAs alter v-ATPase activity in HEK-293T cells.** Cells were first subjected to complete AA starvation for 1 h followed by further culturing for 1 h in either AA-starvation medium ((--)AA), basal medium (all AA at 1* concentration; ++AA), or readdition of each of the individual AA (at 4* concentrations). Then, cells were subjected to the [^3^H]CHLQ accumulation assay. n=7. Bar values are means ± SEM. **p*<0.05 was considered statistically significant.

**Supplementary Methods associated with Supplementary Fig. 1**

Culturing of HEK-293T cells

HEK-293T cells were kindly provided by the Department of Clinical Genetics (Maastricht University Medical Center+, Maastricht, The Netherlands) and cultured as previously described ^14^. Briefly, HEK293T cells were cultured in minimal essential medium supplemented with 10% FBS and 1% penicillin/streptomycin, and cells were grown in a humidified incubator at 37°C with 5% CO_2_. To investigate whether the addition of individual AA affects v-ATPase activity, HEK-293T cells were first subjected to complete AA starvation for 1 h followed by AA-starvation medium (DMEM w/o Amino Acids, USBiological, Swampscott, MA) [(--)AA], individual AA readdition for 1 h, or basal medium for 1 h.

**
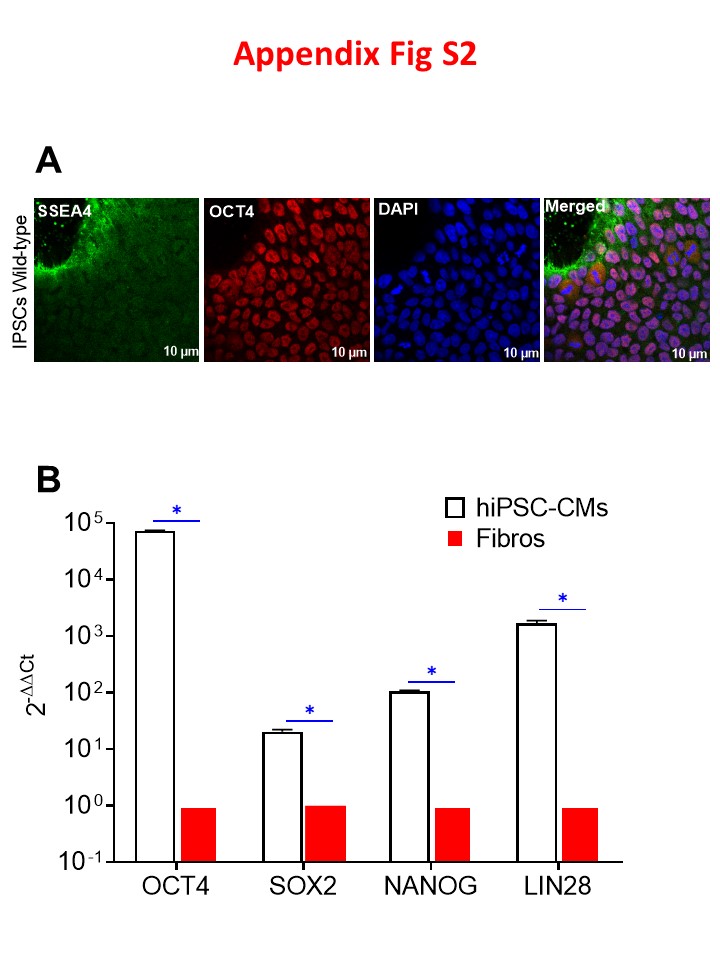
**

**Figure S2 (related to Fig 1D): Characteristics of human-induced pluripotent stem cells differentiated into cardiomyocytes (hiPSC-CMs):** **A.** Expression of pluripotency markers (e.g., SSEA4 and Oct4) and DAPI in hiPSC-CMs, assayed by immunofluorescence microscopy. Scale bar is 10 µm. **B.** Gene expression of pluripotency markers in hiPSCs relative to fibroblasts (n=3). Bar values are means ± SEM. **p*<0.05 were considered statistically significant.

**Supplementary Methods associated with Supplementary Fig. 2**

Immunofluorescent staining for pluripotency markers in hiPSC-CMs

iPSC1700012 (wild type) cells were stained with pluripotent markers SSEA4 and OCT4. iPSC 1700012 (wild type) cell line was fixed with a fixative solution and incubated for 15 min at RT. The staining procedure was performed according to the manufacturer’s instructions using the Pluripotency Stem Cell 4-Market Immunocytochemistry kit (Thermofisher Scientific). Permeabilization solution was added to the cells and incubated for 15 min at RT. Blocking solution was added and incubated for 30 min. The cells were stained with primary antibody (1:100 SSEA4 with 1:200 OCT4 in blocking buffer) for 1 h. Cells were washed with washing buffer and stained with secondary antibody (Alexa Fluor 488 and Alexa Fluor 555) for 1 h at room temperature. Cells were washed with wash buffer and 1 drop of NucBlue Fixed Cell stain (DAPI) was incubated for 5 min. The cells were mounted on a glass slide and imaged at 63x objective with the confocal microscope (Leica SPE). Images were analyzed with ImageJ Fiji.

qPCR analysis for pluripotency markers in hiPSC-CMs

RNA of the samples was isolated using the High pure RNA isolation kit (Roche). After RNA was extracted, cDNA synthesis was performed. The cDNA synthesis was carried out in a total volume of 20 µl containing 1x qScript cDNA supermix (Quantabio) and 500 ng RNA template. PCR program: 5 min at 25°C, 30 min at 42°C, 5 min at 80°C, and hold at 4°C. The qPCR was carried out in a total volume of 10 µl containing 2x Sensimix SYBR Hi-ROX (Bioline), 25 µM primers, and 5 times diluted cDNA. Samples were run on a Lightcycler 480 (Roche) with the following program: 10 min at 95°C and 40 cyclings with 15 sec at 95°C, 15 sec at 60°C, and 15 sec at 72°C, with the relative quantification analysis. Supplementary Table S2 displays primers for pluripotency genes in hiPSC-CMs.

**
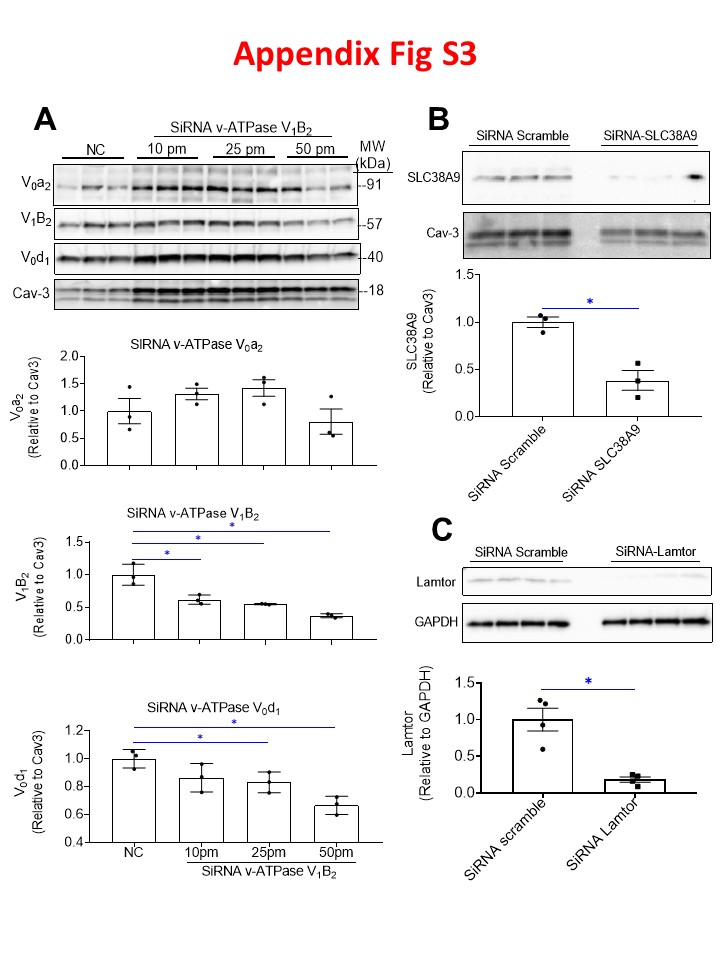
**

**Figure S3: Confirmation of siRNA-mediated silencing of v-ATPase subunit V_1_-B2, lysosomal AA transporter SLC38A9, and Ragulator subunit Lamtor-1 in HL-1 cardiomyocytes.** **A (related to Figure 1B).** Cells were transfected with scrambled siRNA (negative control; NC) or with 10 pm, 25 pm, or 50 pm siRNA targeting v-ATPase B2 subunit RNA. Subsequently, cells were cultured for 32 h. For assessing the efficiency of v-ATPase silencing, V_0_-a2, V_1_-B2, and V_0_-d1 levels were assessed by Western blotting. A representative Western blot of each v-ATPase subunit is displayed. n=3. it should be noted that 50 pm siRNA was used in **Figure 1B**. **B-C (related to Figure 2F).** HL-1 cardiomyocytes were transfected with scrambled siRNA (negative control; SiRNA scramble) or with 75 pm siRNA targeting SLC38A9 RNA and 50 pm siRNA targeting Lamtor-1 RNA. Subsequently, cells were cultured for 32 h. For assessing the efficiency of SLC38A9 and Lamtor-1 silencing, their protein levels were assessed by western blotting. Representative western blots of SLC38A9 and Lamtor-1 are displayed. n=3. Bar values are means ± SEM. **p*<0.05.

**
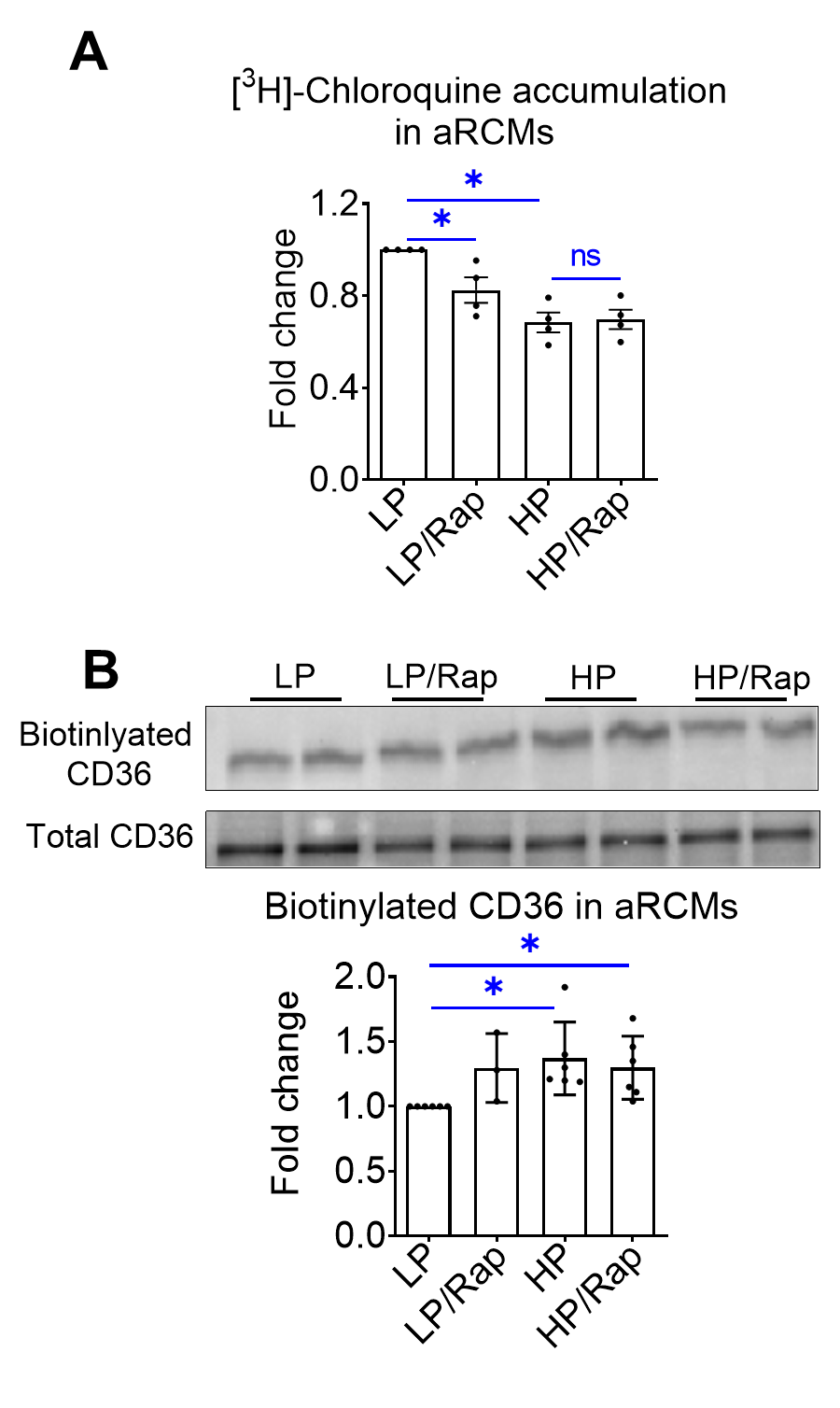
**

**Figure S4: Effects of rapamycin on v-ATPase activation and CD36 translocation in cardiomyocytes under basal conditions and during lipid overexposure.** aRCM were cultured for 24 h under low palmitate (LP) or high palmitate conditions in the absence or presence of 100 nM rapamycin (Rap). **A.** [^3^H]CHLQ accumulation assay for the assessment of v-ATPase activity (n=3). **B.** Representative blots and its quantification cell surface biotinylation assay for measurement of CD36 translocation (n=3-6). Bar values are means ± SEM. **p*<0.05.

**
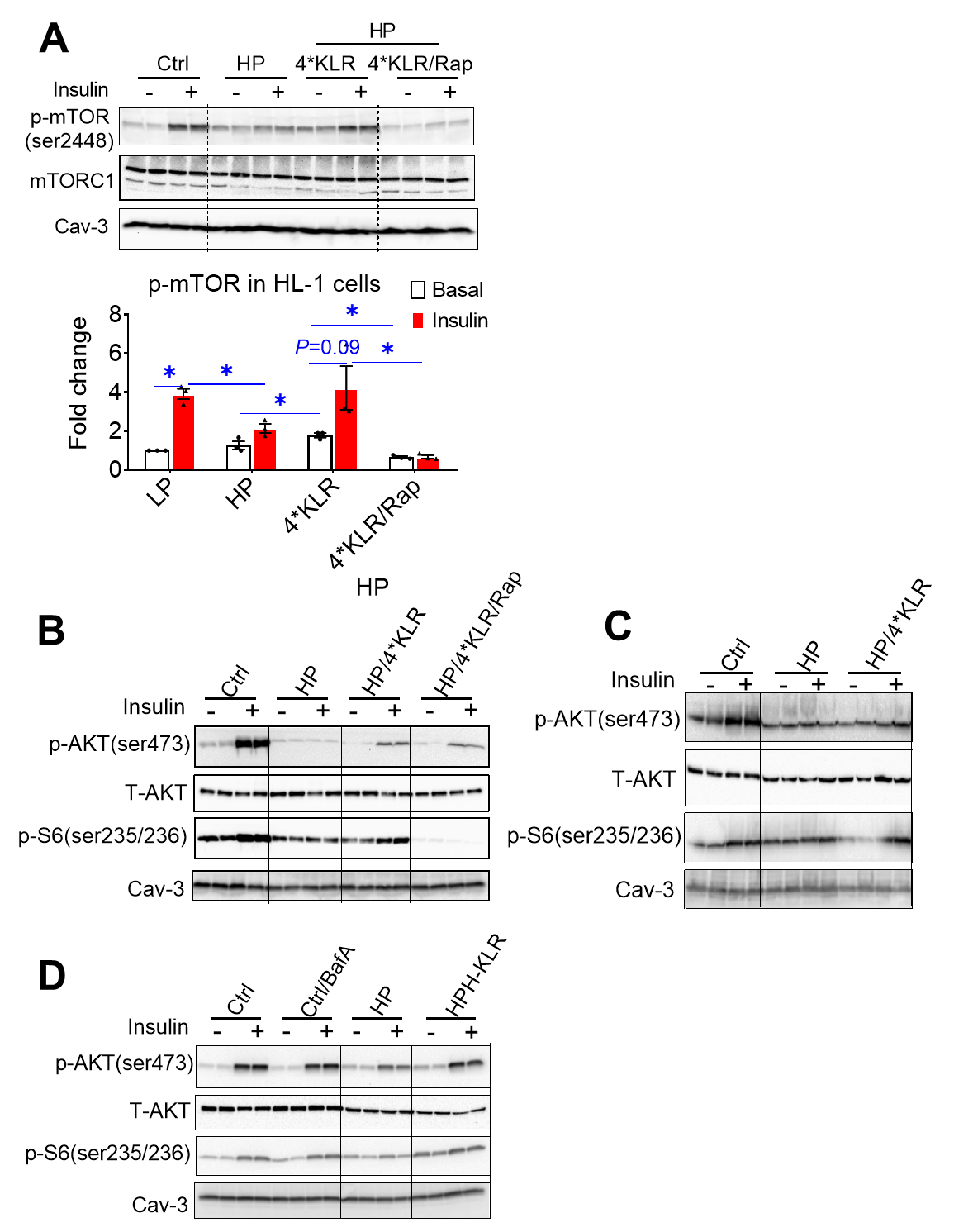
**

**Figure S5: Effects of 4*KLR or H-KLR treatment on insulin signaling in lipid-overexposed cardiomyocytes. A-C.** Effects of 4*KLR treatment **(related to Figure 4A-B).** **A.** mTORC1 activation in HL-1 cells. Cells were cultured for 24 h under various conditions, being Ctrl (no palmitate), high palmitate (HP, palmitate/BSA ratio 3:1), HP supplemented with 4*KLR (HP/4*KLR), or HP/4*KLR supplemented with 100nM rapamycin (HP/4*KLR/Rap). Representative western blots of phospho-mTOR (p-mTOR ser2448), mTORC1 and Cav-3 are displayed. For quantitative comparison of p-mTOR among the different conditions, p-mTOR was normalized against the respective total mTOR signal (n=3). **B-C.** Representative blots of p-AKT (ser473), total-AKT (T-AKT), p-S6 (ser235/236), and Cav-3 (loading control) in HL-1 cells (n=6) and hiPSC-CMs (n=3). **D.** Effects of H-KLR treatment **(related to Figure 5D).** After 23 h culturing under Ctrl or HP conditions and subsequent 1 h H-KLR treatment, HL-1 cells were short-term (30 min) incubated without/with 200 nM insulin. Representative blots of p-AKT (ser473), total-AKT (T-AKT), p-S6 (ser235/236), and Cav-3 (loading control). n=6. Representative Bar values are means ± SEM. **p*<0.05.

**
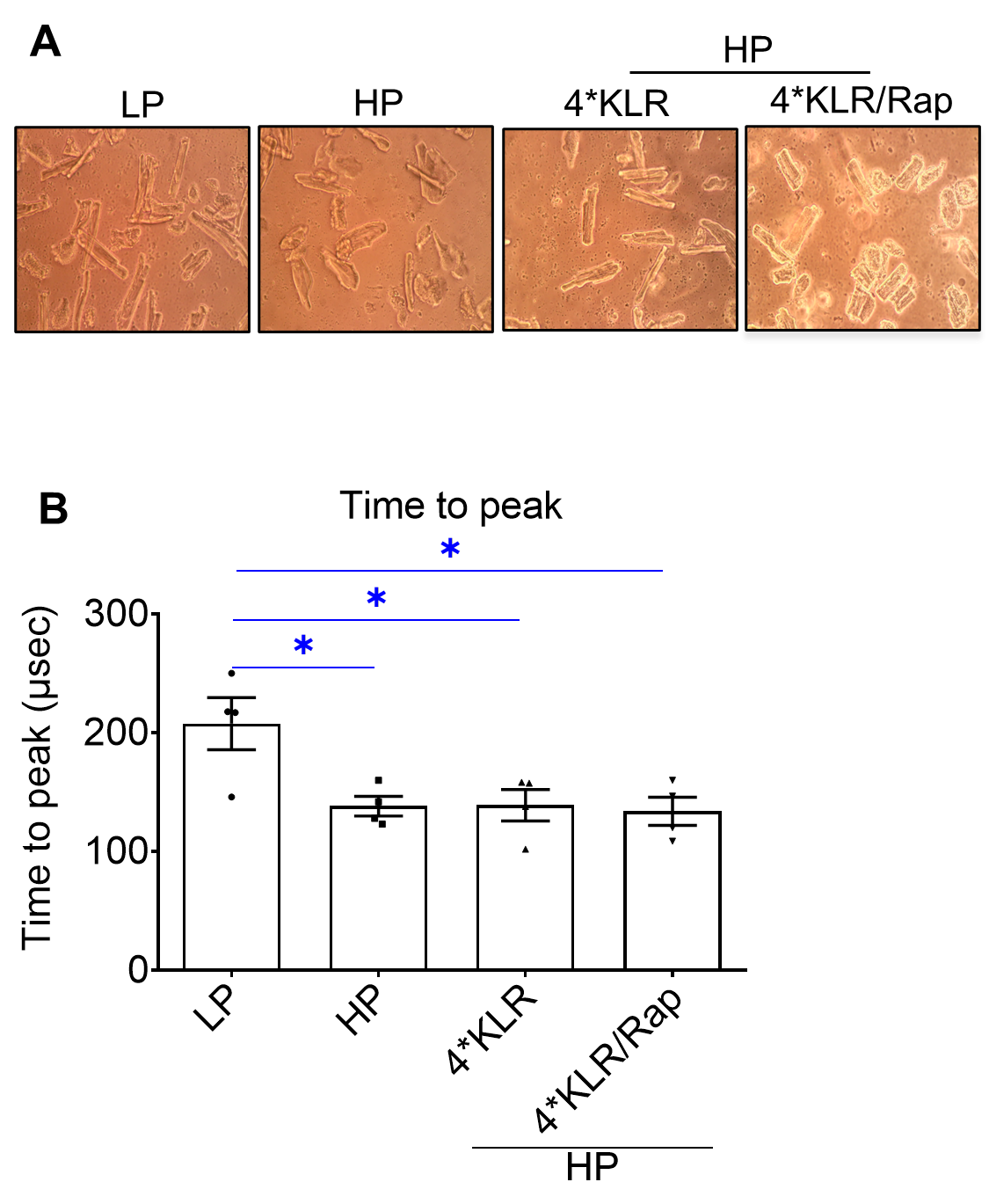
**

**Figure S6 (related to Figure 4J):** **4*KLR treatment prevents contractile dysfunction in lipid-overexposed cardiomyocytes.** aRCMs were cultured for 24 h under the following conditions: low palmitate (LP, basal condition), high palmitate (HP, palmitate/BSA ratio 3:1), HP supplemented with 4*KLR (HP/4*KLR), or HP/4*KLR supplemented with 100 nM Rap (HP/4*KLR/Rap). **A.** At the end of the culturing, images of aRCMs of all tested culturing conditions were taken. Representative pictures are shown. Then, cells were subjected to electric field stimulation at 1 Hz, upon which several contractile parameters were determined, such as sarcomere shortening and decay time (see Figure 4J), and **B.** the time from the onset of the contraction to its peak (time to peak). Bar values are means ± SEM (n=5; imaging of 10 cells/condition). **p*<0.05.

**Figure S7 (related to Figure 5): The 4*KLR cocktail preserves insulin-stimulated glucose uptake in lipid-overexposed cardiomyocytes.** aRCMs were cultured with LP or HP medium for 30 h or for 10 h with HP medium after which the 4*KLR cocktail was added for 0, 1, 3, 10, and 20 h. Subsequently, the cells were stimulated with (-/+) insulin (200 nM) for 30 min and used for the measurement of [^3^H]deoxyglucose uptake. Bar values are means ± SEM (n=3). **p*<0.05.

**
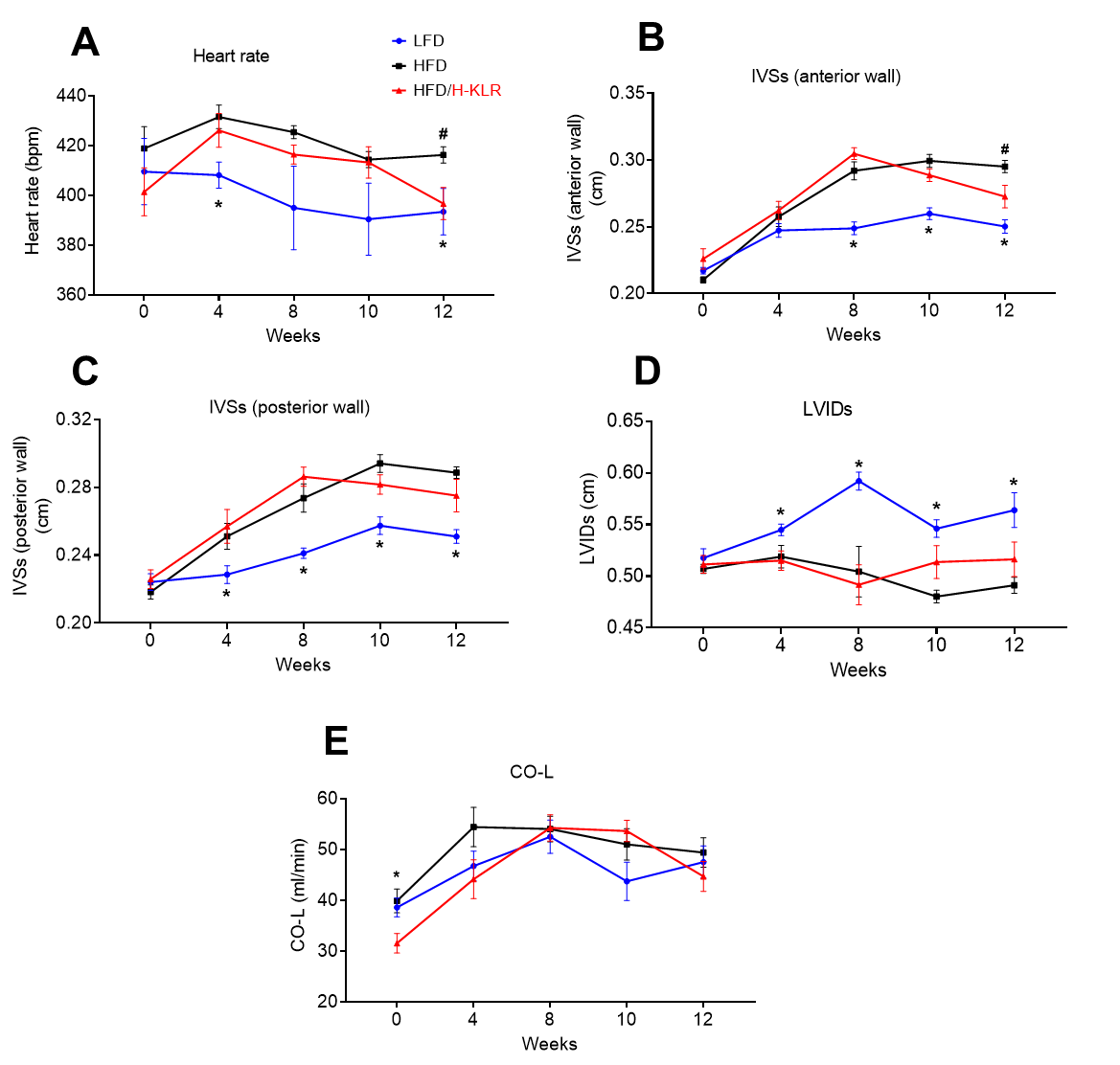
**

**Figure S8: (related to Figure 6B-E)***.* **Effects of H-KLR treatment on echocardiographic parameters in rats fed with a high-fat diet. A-E.** Rats were fed for 12 weeks a low-fat diet (LFD; 10 en% fat), a high-fat diet (HFD; 60 en% fat), or HFD with high concentrations of Lys (7 mM), Leu (12 mM) and Arg (10 mM) added to the drinking water for the last 4 weeks (HFD/H-KLR). Echocardiographic parameters were determined at week 0, 4, 8, 10 and 12: IVSs = Interventricular septal end systole; LVIDs = Left ventricular internal diameter in end systole; CO-L =Cardiac output left ventricular. Graph values are means ± SEM (n=6-7). **p*<0.05 LFD rats vs HFD rats, ^#^*p*<0.05 HFD rats vs HFD/H-KLR rats.

**
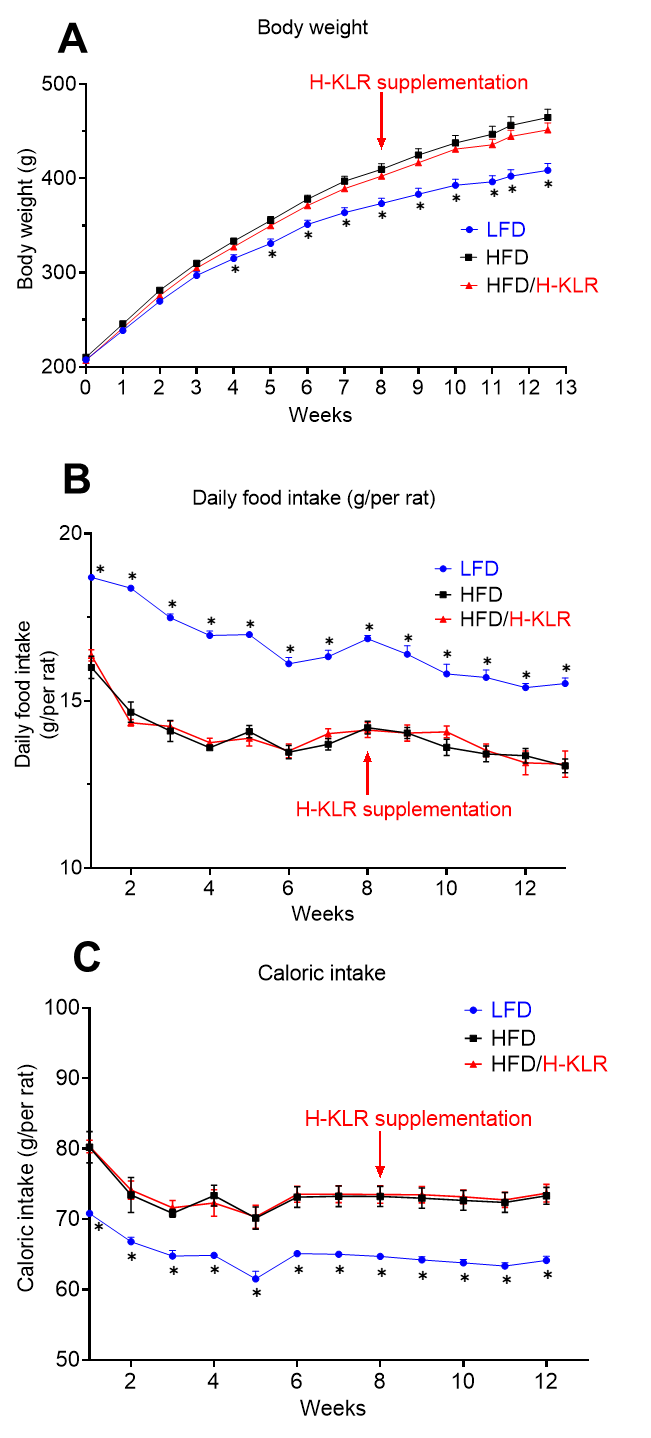
**

**Figure S9: Effects of H-KLR treatment on body weight and feeding patterns in rats fed a high-fat diet.** Rats were fed for 12 weeks a low-fat diet (LFD; 10 en% fat), a high-fat diet (HFD; 60 en% fat), or HFD with high concentrations of Lys (7 mM), Leu (12 mM), and Arg (10 mM) added to the drinking water for the last 4 weeks (HFD/H-KLR). Rats were subjected each week to the measurement of **A*.*** body weight, **B.** daily food intake, and **C.** caloric intake. Values are means ± SEM (n=8). **p*<0.05.

**Table S1: Reagents and resources used in this study**

| REAGENT or RESOURCE | SOURCE | IDENTIFIER |
| --- | --- | --- |
| Antibodies | | |
| phospho-Akt (ser473) | Cell signaling | Catalog#9271 |
| Total-AKT | Cell signaling | Catalog#9272 |
| phospho-mTOR (ser2448) | Cell signaling | Catalog#2971 |
| Total-mTOR | Cell signaling | Catalog#2972 |
| phospho-S6 (ser235/236) | Cell signaling | Catalog#4856 |
| Insulin-regulated aminopeptidase (IRAP) | Cell signaling | Catalog#MABN483 |
| GAPDH (14C10) | Cell signaling | Catalog#2118 |
| Anti-ATP6V_0_D1 (V0d1) | Abcam | Catalog#ab202899 |
| Anti-ATP6V_1_B2 (V1B2) | Abcam | Catalog#ab73404 |
| Anti-ATP6V0A2 (V0-a2) | Abcam | Catalog#ab82638 |
| HA-Tag (C29F4) | Abcam | Catalog#3724(S) |
| CD36 (MO25) | Gift from Dr.N.Tandon | N/A |
| GLUT4 | Millipore | Catalog#07-1404 |
| Caveolin-3 | BD transduction Laboratories | Catalog#610421 |
| SLC38A9 | Abcam | Catalog#ab81687 |
| LAMTOR1 | Abcam | Catalog#ab121157 |
| phospho-ERK1/2 Thr202/Tyr204 | Cell signaling | Catalog#9101 |
| phospho-SAPK/JNK1/2 Thr183/Tyr185 | Cell signaling | Catalog#9251 |
| phospho-4EBP1 | Cell signaling | Catalog#2855 |
| SSEA4 | Thermo Fisher Scientific | Catalog#MA1-021 |
| OCT-4 | Thermo Fisher Scientific | Catalog#MA1–104 |
| Donkey anti-Rabbit IgG (H+L) Highly Cross-Adsorbed Secondary Antibody, Alexa Fluor 594 | Thermo Fisher Scientific | Catalog#A-21207 |
| Donkey anti-Rabbit IgG (H+L) Highly Cross-Adsorbed Secondary Antibody, Alexa Fluor 488 | Thermo Fisher Scientific | Catalog#A32790 |
| Donkey anti-Rabbit IgG (H+L) Highly Cross-Adsorbed Secondary Antibody, Alexa Fluor 555 | Thermo Fisher Scientific | Catalog#A-31572 |
| Anti-rabbit IgG, HRP-linked Antibody | Cell signaling | Catalog#7074 |
| Critical Chemicals, Peptides, and Recombinant Proteins | | |
| [1-^14^C]palmitic acid | GE Healthcare | Catalog#NEC075H250UC |
| 2-Deoxy-d-[1-^3^H]glucose | GE Healthcare | Catalog#NET528A250UC |
| [^3^H]chloroquine | Moravek Biochemicals | Catalog#MT1885 |
| Laminin | Sigma-Aldrich | Catalog#L2020 |
| Insulin | Sigma-Aldrich | Catalog#I6634 |
| Bovine serum albumin (BSA) | MP Biomedicals | Catalog#10735094001 |
| All amino acids | Sigma-Aldrich | N/A |
| Bafilomycin | Sigma-Aldrich | Catalog#11707 |
| Rapamycin | Sigma-Aldrich | Catalog#R8781 |
| Sulfo-NHS-LC-biotin | Thermo Fisher Scientific | Catalog#PG82075 |
| Penicillin-Streptomycin | Thermo Fisher Scientific | Catalog#15070063 |
| Collagenase type II | Worthington | Catalog#NC9693955 |
| Paraformaldehyde | EMS | Catalog#19208 |
| Protein G Sepharose® 4 Fast Flow | GE Healthcare | Catalog#GE17-0618-01 |
| PierceTM high capacity streptavidin beads | Thermo Fisher Scientific | Catalog#20353 |
| Critical Commercial Assays | | |
| Triglyceride Assay Kit | Abcam | Catalog#ab65336 |
| Insulin Rat ELISA Kit | Thermo Fisher Scientific | Catalog#ERINS |
| Pluripotency Stem Cell 4-Market Immunocytochemistry kit | Thermo Fisher Scientific | Catalog#A24881 |
| Lipofectamine™ RNAiMAX | Thermo Fisher Scientific | Catalog number: 1377807 |
| Experimental Models: Cell Lines | | |
| HEK293 | Mouse kindney | N/A |
| HL-1 cells | W.Claycomb, Louisiana State University | N/A |
| Primary adult rat cardiomyocytes | Male Lewis rats | N/A |
| hiPSC-CMs | Skin fibroblasts from healthy adult male individuals | N/A |
| Experimental Models: Organisms/Strains | | |
| Male Lewis rats | Charles River laboratories | N/A |
| Oligonucleotides | | |
| Scramble SiRNA (negative control) | Thermo Fisher Scientific | Catalog#AM4611 |
| V-ATPase B2 (V1B2) SiRNA | Thermo Fisher Scientific | Catalog #AM16708 |
| SLC38A9 SiRNA | Santa Cruz Biotechnology | Catalog#sc-153554 |
| Lamtor SiRNA | Santa Cruz Biotechnology | Catalog#sc-108727 |
| Primers for mRNA expression | See Table S4 | N/A |
| Software and Algorithms | | |
| Prism (version 8) | GraphPad | N/A |
| Image J/Fiji | Fiji | https://imagej.nih.gov/ij/ |
| Other |  |  |
| Microvette CB 300 Z | SARSTEDT | Catalog#16.440.100 |
| Glucose meters | Bayer Contour XT | ASCENSIA Diabetes Care |
| Nitrocellulose membrane (0.45 mm pore) | Invitrogen | Catalog#LC2001 |

**Table S2: Primers for pluripotency genes in human-induced pluripotent stem cells differentiated into cardiomyocytes (hiPSC-CMs).**

| **Pluripotency genes** | **Species** | **Primer sequence** | **Melting temperature (°C)** | **Fwd/Rev** |
| --- | --- | --- | --- | --- |
| SOX2 | Human | CCCAGCAGACTTCACATGT | 60 | fwd |
| SOX2 | Human | CCTCCCATTTCCCTCGTTTT | 60 | rev |
| OCT4 | Human | CCTCACTTCACTGCACTCTA | 60 | fwd |
| OCT4 | Human | CAGGTTTTCTTTCCCTAGCT | 60 | rev |
| NANOG | Human | CTCCATGAACATGCAACCTG | 60 | fwd |
| NANOG | Human | GGCATCATGGAAACCAGAAC | 60 | rev |
| LIN28 | Human | CACAGGGAAAGCCAACCTAC | 60 | fwd |
| LIN28 | Human | TGCACCCTATTCCCACTTTC | 60 | rev |
| ACTA2 | Rat | AAGGCCAACCGGGAGAAAAT | 60 | fwd |
| ACTA2 | Rat | AGTCCAGCACAATACCAGTTG | 60 | rev |
| CTGF | Rat | CACAGAGTGGAGCGCCTGTTC | 60 | fwd |
| CTGF | Rat | GATGCACTTTTTGCCCTTCTTAATG | 60 | rev |
| Col1A1 | Rat | GAGTACTTCTGTAAGGCGCAG | 60 | fwd |
| Col1A1 | Rat | GGCATTCTGGAAGCAATCATG | 60 | rev |
| CILP1 | Rat | GAGTACTTCTGTAAGGCGCAG | 60 | fwd |
| CIPL1 | Rat | GGCATTCTGGAAGCAATCATG | 60 | rev |
| ANP | Rat | ATCACCAAGGGCTTCTTCCT | 60 | fwd |
| ANP | Rat | TGTTGGACACCGCACTGTAT | 60 | rev |
| Cyclo | Rat | CAAATGCTGGACCAAACACAA | 60 | fwd |
| Cyclo | Rat | TTCACCTTCCCAAAGACCACAT | 60 | rev |

**Note:** Alpha-Smooth muscle actin (ACTA2); Connective tissue growth factor (CTGF); Collagen, type 1, alpha 1 (Col1A1); Cartilage intermediate-layer protein 1 (CILP1); Atrial natriuretic peptide (ANP); Cyclophilin-A (Cyclo; housekeeping gene).

**Table S3: Concentrations of the individual acids in the 4*KLR and H-KLR cocktails**

| Amino acid | 4 x physiological concentration (mM) | High physiological  Concentration (mM) |
| --- | --- | --- |
| Lys (K) | 1.56 | 7 (1) |
| Leu (L) | 1.84 | 12 (2) |
| Arg (R) | 1.36 | 10 (3) |
| Thr (T) | 1.80 |  |
| Gln (Q) | 10.00 |  |
| Ile (I) | 1.66 |  |
| Gly (G) | 1.00 |  |
| Ser (S) | 1.00 |  |
| Phe (F) | 0.86 |  |
| Asn (N) | 0.20 |  |
| Asp (D) | 0.20 |  |
| Cys (C) | 0.40 |  |
| Ala (A) | 0.20 |  |
| His (H) | 0.60 |  |
| Met (M) | 0.46 |  |
| Pro (P) | 0.60 |  |

**References:**

1. Layman, D. K., & Walker, D. A. (2006). Potential importance of leucine in treatment of obesity and the metabolic syndrome. The Journal of Nutrition, 136(1), 319S-323S.
2. Piatti, P., Monti, L. D., Valsecchi, G., Magni, F., Setola, E., Marchesi, F., ... & Alberti, K. G. M. (2001). Long-term oral L-arginine administration improves peripheral and hepatic insulin sensitivity in type 2 diabetic patients. Diabetes Care, 24(5), 875-880.
3. Kim, I. Y., Williams, R. H., Schutzler, S. E., Lasley, C. J., Bodenner, D. L., Wolfe, R. R., & Coker, R. H. (2014). Acute lysine supplementation does not improve hepatic or peripheral insulin sensitivity in older, overweight individuals. Nutrition & Metabolism, 11(1), 49-49.

**Table S4: Effects of H-KLR treatment on organ/tissue parameters in rats fed a high-fat diet**

|  | LFD | HFD | HFD/H-KLR |
| --- | --- | --- | --- |
|  | Week 12 (Mean ± SEM) | | |
| Tibial length (g) | 4.08±0.019 | 4.074±0.019 | 4.056±0.012 |
| Heart weight/Tibial length | 1.00±0.01 | 1.04±0.03 | 1.00±0.01 |
| Gastrocnemius/Tibial length | 1.00±0.01 | 1.05±0.01^a^ | 1.03±0.02^b^ |
| Quadriceps/Tibial length | 1.00±0.06 | 1.21±0.03^a^ | 1.15±0.03^b^ |
| Tibialis anterior/Tibial length | 1.00±0.10 | 1.09±0.05 | 1.07±0.06 |
| Soleus/Tibial length | 1.00±0.04 | 1.06±0.04 | 1.00±0.02 |
| Kidney/Tibial length | 1.00±0.02 | 1.03±0.02 | 1.01±0.01 |

Rats were fed for 12 weeks a low-fat diet (LFD; 10 en% fat), a high-fat diet (HFD; 60 en% fat), or HFD with high concentrations of Lys (7 mM), Leu (12 mM), and Arg (10 mM) added to the drinking water for the last 4 weeks (HFD/H-KLR). At the end of the 12 weeks diet regime, organ/tissue parameters were measured. Except for tibia length, values have been normalized to the corresponding LFD conditions. All values are expressed as means ± SEM (n=6-8). ^a^ Significantly different from LFD (*P*<0.05). ^b^ Significantly different from HFD (*P*<0.05).

**Table S5: Effects of H-KLR treatment on cardiac fibrosis in rats fed a high-fat diet**

|  | LFD | HFD | HFD/H-KLR |
| --- | --- | --- | --- |
|  | Week 12 (Mean ± SEM) | | |
| ACTA2 | 1.00±0.16 | 0.87±0.09 | 0.88±0.06 |
| CTGF | 1.00±0.09 | 0.69±0.08^a^ | 0.79±0.08 |
| Col1a1 | 1.00±0.08 | 0.97±0.07 | 0.84±0.07 |
| CILP1 | 1.00±0.04 | 0.91±0.12 | 1.03±0.31 |
| ANP | 1.00±0.48 | 3.86±2.24 | 0.65±0.25 |

Rats were fed for 12 weeks a low-fat diet (LFD; 10 en% fat), a high-fat diet (HFD; 60 en% fat), or HFD with high concentrations of Lys (7 mM), Leu (12 mM), and Arg (10 mM) added to the drinking water for the last 4 weeks (HFD/H-KLR). At the end of the 12 weeks diet regime, markers of fibrosis and hypertrophy were measured by RT-PCR. Smooth muscle actin, ACTA2; Connective tissue growth factor, CTGF; Collagen, type 1, α1, Col1A1; Cartilage intermediate-layer protein-1, CILP1; Atrial natriuretic peptide, ANP. Values are means ± SEM (n=8). ^a^ Significantly different from LFD (*P*<0.05). ^b^ Significantly different from HFD (*P*<0.05).

**Supplementary Methods associated with Supplementary Table. S5**

RT-PCR analysis for fibrosis/hypertrophy markers

Total RNA was isolated from cardiac tissue samples using RNeasy kits (Qiagen) and reversed transcribed into cDNA using the iScript cDNA synthesis kit (Biorad) according to the manufacturer’s protocol. Real-time PCR was performed on an iCycler accompanied by the My IQ single color real-time PCR detection system using iQ SYBR-Green Supermix (Biorad). Gene expression levels of the fibrosis/hypertrophy markers were normalized using the housekeeping gene Cyclophilin-A, and their relative expression was calculated using the comparative threshold cycle (Ct) method.

**Table S6: Effects of H-KLR supplementation of plasma AA concentration in rats**

|  | LFD | HFD | HFD/H-KLR |
| --- | --- | --- | --- |
|  | Week 12 (Mean ± SEM) | | |
| ASN | 80.35±6.65 | 104.32±7.94^a^ | 89.65±9.08^b^ |
| SER | 244.05±19.45 | 308.69±10.38^a^ | 292.31±15.47^b^ |
| GLN | 791.25±57.55 | 1037.95±26.63^a^ | 919.39±25.89^a,b^ |
| HIS | 74.78±4.05 | 97.13±2.88^a^ | 86.28±2.97^a,b^ |
| GLY | 232.87±13.51 | 316.42±7.48^a^ | 285.14±17.39^b^ |
| THR | 378.50±25.73 | 424.79±7.74^a^ | 370.35±19.62^b^ |
| CIT | 73.42±4.14 | 90.03±4.00^a^ | 87.93±2.98^a^ |
| ARG | 150.05±20.02 | 165.98±17.43 | 208.67±13.57^a,b^ |
| ALA | 436.16±16.35 | 507.11±17.14^a^ | 441.41±26.48^b^ |
| TAU | 289.51±17.07 | 314.32±16.70 | 294.17±19.19 |
| AIB | 17.01±1.89 | 25.80±1.34^a^ | 23.58±2.30^a^ |
| TYR | 135.46±5.87 | 157.09±7.83^a^ | 150.41±8.45^a^ |
| VAL | 265.82±13.22 | 308.68±6.49^a^ | 281.94±12.15^b^ |
| MET | 74.19±3.99 | 95.92±3.14^a^ | 87.92±4.08^a^ |
| NVAL | 506.63±2.61 | 521.54±5.30^a^ | 504.07±4.07^b^ |
| ILE | 120.15±6.50 | 139.66±4.59^a^ | 129.84±5.86^b^ |
| PHE | 79.04±4.59 | 86.92±2.56 | 84.90±2.05 |
| TRP | 60.07±8.57 | 57.06±4.63 | 48.41±4.34 |
| LEU | 199.12±9.69 | 228.32±6.31^a^ | 229.99±7.50^a^ |
| ORN | 133.59±9.15 | 137.60±21.73 | 94.07±5.82^b^ |
| LYS | 545.13±33.68 | 700.40±33.77^a^ | 720.82±37.97^a^ |

Rats were fed for 12 weeks a low-fat diet (LFD; 10 en% fat), a high-fat diet (HFD; 60 en% fat), or HFD with high concentrations of Lys (7 mM), Leu (12 mM), and Arg (10 mM) added to the drinking water for the last 4 weeks (HFD/H-KLR). At the end of the 12 weeks diet regime, plasma AA levels were measured. Plasma concentrations of Lys, Leu, and Arg are highlighted in yellow. Values are means ± SEM (n=6). ^a^ Significantly different from LFD (*P*<0.05). ^b^ Significantly different from HFD (*P*<0.05).
